# Structure–Activity Relationship of Cycling Molecular Assemblies for Golgi Targeting and Disruption

**DOI:** 10.1021/acs.jmedchem.6c01269

**Published:** 2026-07-08

**Authors:** Weiyi Tan, Qiuxin Zhang, Pengyu Hong, Bing Xu

**Affiliations:** † Department of Chemistry, 8244 Brandeis University, Waltham, Massachusetts 02453, United States; ‡ Department of Computer Science, Brandeis University, Waltham, Massachusetts 02453, United States

## Abstract

Targeting the Golgi apparatus with small molecules remains challenging because of limited binding targets and poor selectivity in cells. We recently introduced cycling molecular assemblies (CyMAs), which use a palmitoylation/depalmitoylation enzyme switch to kinetically trap supramolecular nanostructures at the Golgi, but the molecular features governing their activity were unclear. Here, we report a structure–activity relationship (SAR) study of CyMA by systematically varying the peptide backbone, thioester warhead, and N-terminal capping group. Analysis of Golgi localization, trapping kinetics, critical aggregation concentration (CAC), and cytotoxicity revealed that the self-assembling ability is the primary determinant of CyMA activity. The d-Phe-d-Phe backbone exhibits optimal assembly and enables efficient Golgi accumulation at nanomolar concentrations. Warhead and N-terminal modifications further tune trapping kinetics and disrupt potency. Together, these results define design principles for CyMA and establish enzyme-driven self-assembly as a general, modular strategy for developing drugs for Golgi and organelle targeting.

## Graphical abstract

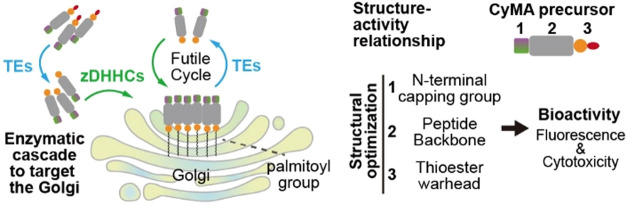

## Introduction

The Golgi apparatus is a central hub for protein and lipid processing, post-translational modification, and intracellular trafficking.^1–6^ By regulating biosynthetic maturation and spatial deployment of signaling proteins, the Golgi contributes directly to the functional output of disease-relevant pathways. For example, the processing or trafficking of proteins such as RAS,^7^ EGFR,^8^ and TGFB1,^9^ and immune checkpoint proteins including PD-L1 ^10^ depend on Golgi-associated machinery. Because of these roles, the selective targeting of the Golgi could provide useful opportunities for live-cell imaging and therapeutic intervention. However, achieving precise Golgi localization with small molecules remains difficult because of drug efflux. Conventional approaches often lack organelle selectivity, rely on poorly defined localization motifs, or require prolonged incubation,^11–14^ and many current targeting agents are better suited to fixed-cell studies than to dynamic interrogation in living systems.^15^

To address this challenge, we recently developed cycling molecular assemblies (CyMAs),^16^ a molecular platform that advances the paradigm of enzyme-instructed self-assembly (EISA).^17–30^ This strategy hijacks an endogenous palmitoylation/depalmitoylation enzyme switch, which is composed of Golgi-resident palmitoyl acyltransferases (zDHHCs)^31,32^ and intracellular acyl-protein thioesterases (TEs),^33^ to drive a futile acylation cycle that results in the active, nonequilibrium sequestration of supramolecular nanostructures at the Golgi.^16^ While our initial discovery established that this cycling-and-trapping mechanism enables rapid Golgi imaging and functional disruption^16,34–36^ without relying on conventional ligand–receptor recognition,^37^ the fundamental molecular rules governing this unique behavior remained unexplored.

Because CyMA function arises from dynamic enzymatic turnover coupled to in situ self-assembly rather than from static target occupancy, optimizing this platform requires a structure–activity relationship (SAR) framework distinct from that used for classical ligand-based drug design. To establish quantitative design principles, we constructed a library of over 40 precursors (**1b**–**1w**, **3b**–**3r**, and **4a**–**4c**) based on three distinct structural rationales (Scheme 1): (i) The N-terminal capping group (NTG). We modulated the NTG from compact, environment-sensitive fluorophores (for imaging applications, CyMA-i) to rigid, extended multiring aromatic architectures (for disruption applications, CyMA-d).^16,34,38^ (ii) The peptide backbone. We systematically varied the dipeptide core across aliphatic, polar, charged, and aromatic configurations, alongside homochiral and heterochiral stereochemical variations. The rationale was to map how side-chain hydrophobicity, directional hydrogen bonding, and aromatic π-π stacking density collectively dictate the critical aggregation concentration (CAC),^39,40^ thereby determining the thermodynamic threshold required for assembly. (iii) The thioester warhead. We engineered the warhead with diverse acyl modifications, systematically altering aliphatic chain lengths, branching, and electronic profiles.^35^ Following cellular entry, the dynamic processing of these components determines targeting efficiency. The precursors are cleaved by intracellular thioesterases, including PPT1,^42^ LYPLA1,^43^ and LYPLA2,^44^ to yield free-thiol intermediates, which are subsequently S-palmitoylated by Golgi-resident zDHHC enzymes using endogenous palmitoyl-CoA. This acylation generates an amphiphilic species with an enhanced propensity to self-assemble, converting transient enzymatic cycling into stable, nondiffusive kinetic trapping at the Golgi.^16^

**Scheme 1:**
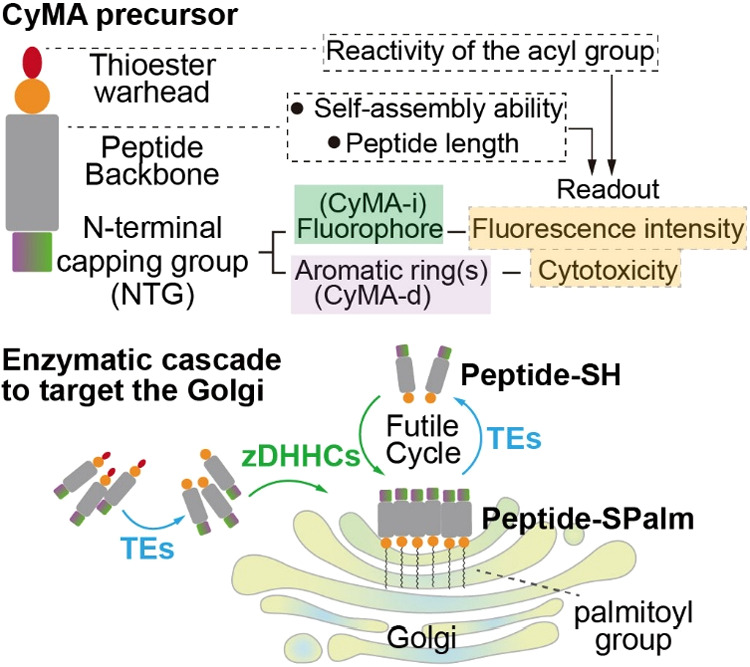
Building Blocks of the CyMA Precursor and the Enzymatic Cascade for Golgi Targeting by CyMA

Building upon this modular design, here we present a comprehensive profiling of the expanded library utilizing Golgi fluorescence intensity, trapping kinetics, critical aggregation concentration (CAC), and cytotoxicity as integrated cellular and biophysical readouts. Our systematic analysis demonstrates that while processing kinetics and structural characteristics influence the system, thermodynamic self-assembling ability serves as a foundational driver governing CyMA performance and organelle localization efficiency. In particular, the d-Phe-d-Phe backbone provides a highly favorable balance of assembly propensity and cell entry, enabling efficient Golgi accumulation even at nanomolar concentrations, whereas variants lacking an adequate assembling ability exhibit less efficient retention and tend to remain in a predominantly diffusive state. These results establish clear physicochemical design principles for CyMA and provide a valuable framework for developing enzyme-switched molecular assemblies as nondiffusive therapeutics for organelle-targeted drugs.

## Results and Discussion

### Molecular Design

To define the structural features that govern CyMA activity, we designed analogues based on the previously reported CyMA-i scaffold **1a** and CyMA-d scaffold **3a**.^16^ In **1a**, an NBD fluorophore serves as the N-terminal capping group (NTG), the peptide backbone is d-Phe-d-Phe, and the C-terminus contains a methyl thioester. Because CyMA activity is expected to depend on the interplay of self-assembly, enzymatic activation, and NTG-dependent output, we varied each of these structural elements systematically (Scheme 2).

**Scheme 2:**
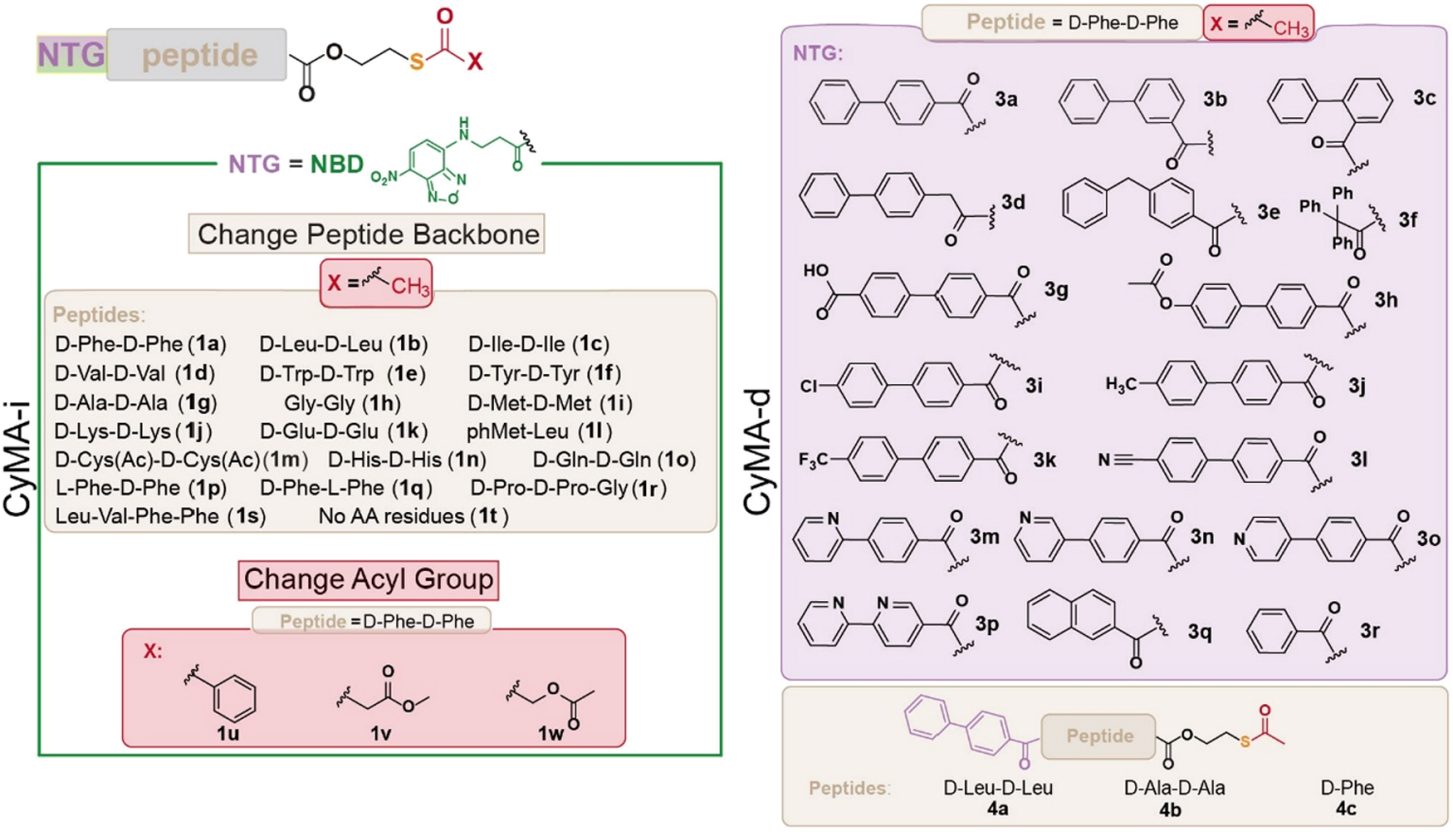
Molecular Structures of CyMA Precursors, Including CyMA-i (Left Panel) and CyMA-d (Right Panel)

For the imaging series (CyMA-i), we primarily modified the peptide backbone of **1a** to examine how the residue identity, stereochemistry, charge, and molecular length affect Golgi targeting. The d-Phe-d-Phe sequence was replaced with a range of dipeptides, including D-dileucine (**1b**), D-diisoleucine (**1c**), D-divaline (**1d**), D-ditryptophan (**1e**), D-dityrosine (**1f**), D-dialanine (**1g**), diglycine (**1h**), D-dimethionine (**1i**), D-dilysine (**1j**), D-diglutamate (**1k**), L-photomethionine-l-leucine (**1l**), D-di-S-acetylcysteine (**1m**), D-dihistidine (**1n**), and D-diglutamine (**1o**). To assess whether CyMA localization depends on specific stereochemical recognition, we also synthesized heterochiral analogues **1p** (l-Phe-d-Phe) and **1q** (d-Phe-l-Phe). In addition, tripeptide and tetrapeptide analogues **1r** and **1s** were synthesized to evaluate the effect of increased molecular length, and a truncated analogue **1t**, which lacks amino acid residues, was included as a mechanistic control. Finally, we varied the thioester warhead to probe the effect of the acyl structure on enzymatic cycling by preparing the benzoyl (**1u**), 3-methoxy-3-oxopropanoyl (**1v**), or acetoxyacetyl (**1w**) analogues.

For the disruption series (CyMA-d), we replaced the biphenyl NTG in **3a** with a set of regioisomeric, substituted, and heteroaromatic motifs to generate precursors **3b**–**r**. To test whether cytotoxicity requires the same assembly-driving backbone identified in the imaging series, we also attenuated the self-assembling ability of **3a** by replacing d-Phe-d-Phe with dileucine, dialanine, or phenylalanine, yielding analogues **4a**–**4c**. Together, this library helps distinguish the roles of the peptide backbone, thioester warhead, and NTG in determining Golgi localization, trapping kinetics, and biological activity.

### Synthesis of CyMA Precursors

We synthesized the CyMA precursors using an Fmoc-based solid-phase peptide synthesis (SPPS) strategy (Scheme S2). We first functionalized the 2-chlorotrityl chloride resin with 2-mercaptoethanol, which attached selectively through its thiol group because the thiol is more nucleophilic than the hydroxyl group. We then installed the first Fmoc-protected amino acid by esterifying the resin-bound alcohol with its C-terminal carboxylate to form the depsipeptide linkage, and we elongated the peptide by standard SPPS protocols.

After completing peptide assembly, we capped the N-terminus on resin with the appropriate NTG, including NBD for CyMA-i analogues and biphenyl-derived aromatic motifs for CyMA-d analogues. We cleaved the product from the resin with a TFA-based cocktail to afford the free-thiol intermediate and then converted it to the corresponding thioester with the appropriate acyl chloride using solution-phase synthesis. We purified the final products using reversed-phase HPLC and characterized them by LC-HRMS. Throughout the paper, lowercase letters denote D-amino acid residues, whereas uppercase letters denote L-amino acid residues.

### Peptide Backbone Governs Golgi Localization

We first evaluated the imaging series **1a**–**1w** in HeLa cells using live-cell confocal laser scanning microscopy (CLSM) to determine how the peptide backbone influences Golgi accumulation. The parent CyMA scaffold **1a** contains the d-Phe-d-Phe (ff) motif, which is known to promote supramolecular assembly through hydrophobic and aromatic interactions.^39^ As shown in the CLSM images at 10 μM (Figures 1A and S1), the dipeptide backbone strongly influenced both intensity and selectivity of Golgi fluorescence. The slightly broader spatial profile of **1a** relative to the specific Golgi marker points to the dynamic nature of the platform, where enzymatically activated assemblies participate in continuous membrane cycling and trafficking between the Golgi apparatus and the endoplasmic reticulum (ER).^2^ Analogues containing hydrophobic residues, including **1a** (ff), **1b** (ll), **1c** (ii), **1d** (vv), and **1e** (ww), exhibited intense Golgi-localized fluorescence within 16 min (Figures 1B and S2–S6). These results indicate that hydrophobic backbones can support efficient cycling-and-trapping, presumably by favoring membrane partitioning and subsequent assembly after enzymatic activation.^45^ Among these compounds, however, the aromatic d-Phe-d-Phe scaffold consistently gave the most robust accumulation, suggesting that aromatic–aromatic interactions provide an advantage beyond hydrophobicity alone.

**Figure 1:**
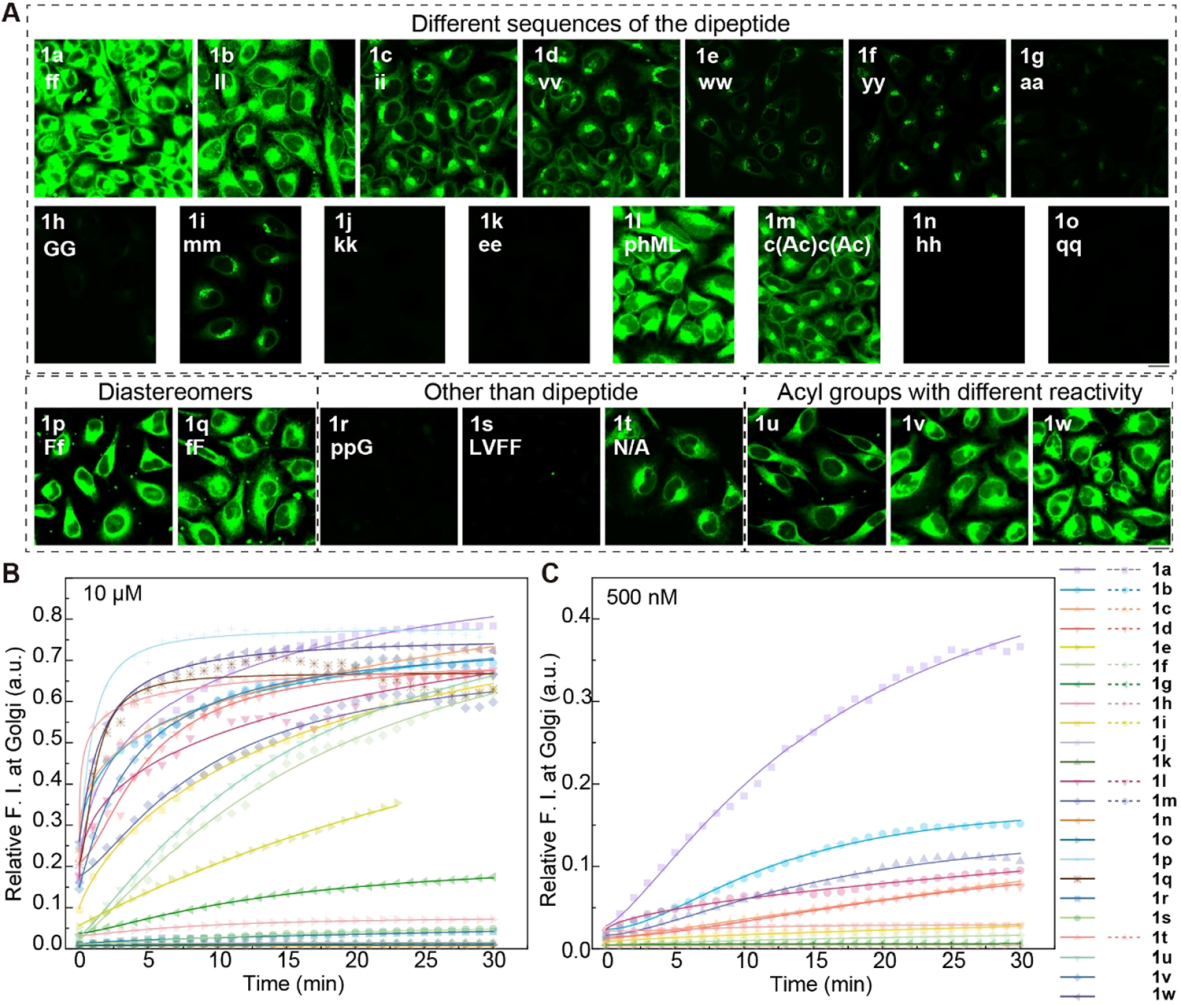
(A) CLSM of HeLa cells treated with various CyMA-i precursors (**1a**–**1w**) (10 μM) for 16 min. (B) Quantitative analysis of fluorescence intensity at the Golgi of HeLa cells treated with **1a**–**1w** at 10 μM (0–30 min). (C) Quantitative analysis of fluorescence intensity at the Golgi of HeLa cells treated with **1a**–**1w** at 500 nM (0–30 min). Scale bar = 20 μm.

In contrast, analogues containing more polar, weakly hydrophobic, or charged residues generally showed an impaired localization. Analogues **1f** (yy), **1g** (aa), and **1h** (GG) exhibited reduced Golgi fluorescence, while **1j** (kk) and **1k** (ee) showed little or no detectable accumulation (Figures 1A,B and S7–S11). Corresponding cell viability evaluations confirmed that these nonresponding variants maintain excellent biocompatibility (Figure S12), ruling out acute toxicity as a cause for the absence of signal and confirming that the cells remain fully capable of driving the enzymatic reactions. These results indicate that the CyMA platform tolerates some variation in the backbone composition, but efficient Golgi targeting requires a sufficiently strong tendency to assemble after enzymatic conversion. For more polar analogues, reduced accumulation may reflect not only weaker self-assembly but also altered intracellular handling, including increased susceptibility to competing chemical or enzymatic processes.^46^

The backbone length also proved to be important. Extension from a dipeptide to a tripeptide or tetrapeptide, as in **1r** and **1s**, reduced targeting efficiency (Figures 1A,B, S13, and S14). Importantly, these larger backbones alter multiple physicochemical variables simultaneously beyond mere molecular length. Specifically, **1r** (d-Pro-d-Pro-Gly) introduces conformationally constrained proline rings alongside a highly flexible glycine hinge, severely disrupting the cooperative aromatic packing observed in the dipeptide series. Meanwhile, **1s** (Leu-Val-Phe-Phe) significantly scales up the local steric bulk and changes the overall hydrophobicity profile. This decrease in organelle retention may arise from poorer steric accessibility to intracellular processing enzymes, slower trafficking, or altered intermolecular packing of the lipidated intermediates, proving that the length operates in strict cross-talk with backbone flexibility and composition. Thus, these results support a relatively narrow structural window in which the precursor remains compatible with both the enzymatic cycling and productive assembly.

The truncated control **1t** provided additional mechanistic insight. Despite lacking a peptide backbone, **1t** entered cells rapidly and showed initial Golgi localization (Figures 1A,B and S15), indicating that the thioester motif alone can support access to the palmitoylation cycle. However, this analogue displays much weaker assembly propensity than the peptide-containing analogues, suggesting that the peptide backbone is not required for initiating Golgi access but is essential for stabilizing nondiffusive accumulation. Its small size and relatively high lipophilicity may also favor passive diffusion across the plasma membrane^47^ rather than the mixed uptake pathways proposed for peptide-bearing CyMA analogues.^16,48–50^

The heterochiral diastereomers **1p** (Ff) and **1q** (fF) showed Golgi-targeting behavior comparable to that of the homochiral parent **1a** (Figures S16 and S17). This absence of stereoselectivity argues against a classical ligand–receptor mechanism, which would typically require more stringent chiral recognition, and instead supports a model in which localization arises primarily from the collective physical properties of supramolecular assemblies associated with enzymatic activation.^51^

### Acyl Group Modulation of Enzyme Cycle Kinetics

We next examined how the thioester warhead affects the rate of Golgi accumulation. Quantitative analysis at 10 μM (Figure 1B) showed that changing the acyl group significantly modulates the rate of fluorescence increase without changing the underlying targeting mechanism. The parent acetyl derivative **1a** and the β-keto ester analogue **1v** both reached steady-state fluorescence rapidly (Figure S18), whereas the benzoyl **1u** accumulated much more slowly (Figure S20). While direct in vitro enzymatic cleavage rates were not explicitly measured in this study, this kinetic profile aligns with literature precedent suggesting that a bulky aromatic ring reduces access of intracellular thioesterases to the scissile thioester bond, thereby slowing entry into the enzymatic cycle.^7,52^

A comparison of **1v** and **1w** further shows that the intrinsic chemical reactivity alone does not predict cellular performance. Although the thioester in **1v** is electronically more activated than in **1w**, **1w** displayed a faster fluorescence turn-on and a shorter apparent *t*_1/2_ in cells (Figures 2A, S18, and S19). Because the observed fluorescence response reflects the combined effects of uptake, organelle access, enzymatic cleavage, palmitoylation, and postconversion assembly, the apparent rate in cells cannot be assigned solely to thioester electrophilicity. Instead, the faster response of **1w** likely reflects better compatibility with intracellular thioesterases, which often favor relatively simple, short-chain acyl motifs.^53^ Thus, while the baseline concept of dynamic acylation is adopted from our earlier platform design,^16^ the present work explicitly establishes that the molecular structure of the warhead primarily tunes the kinetics of intracellular activation, whereas successful organelle trapping remains contingent on downstream supramolecular assembly.

**Figure 2:**
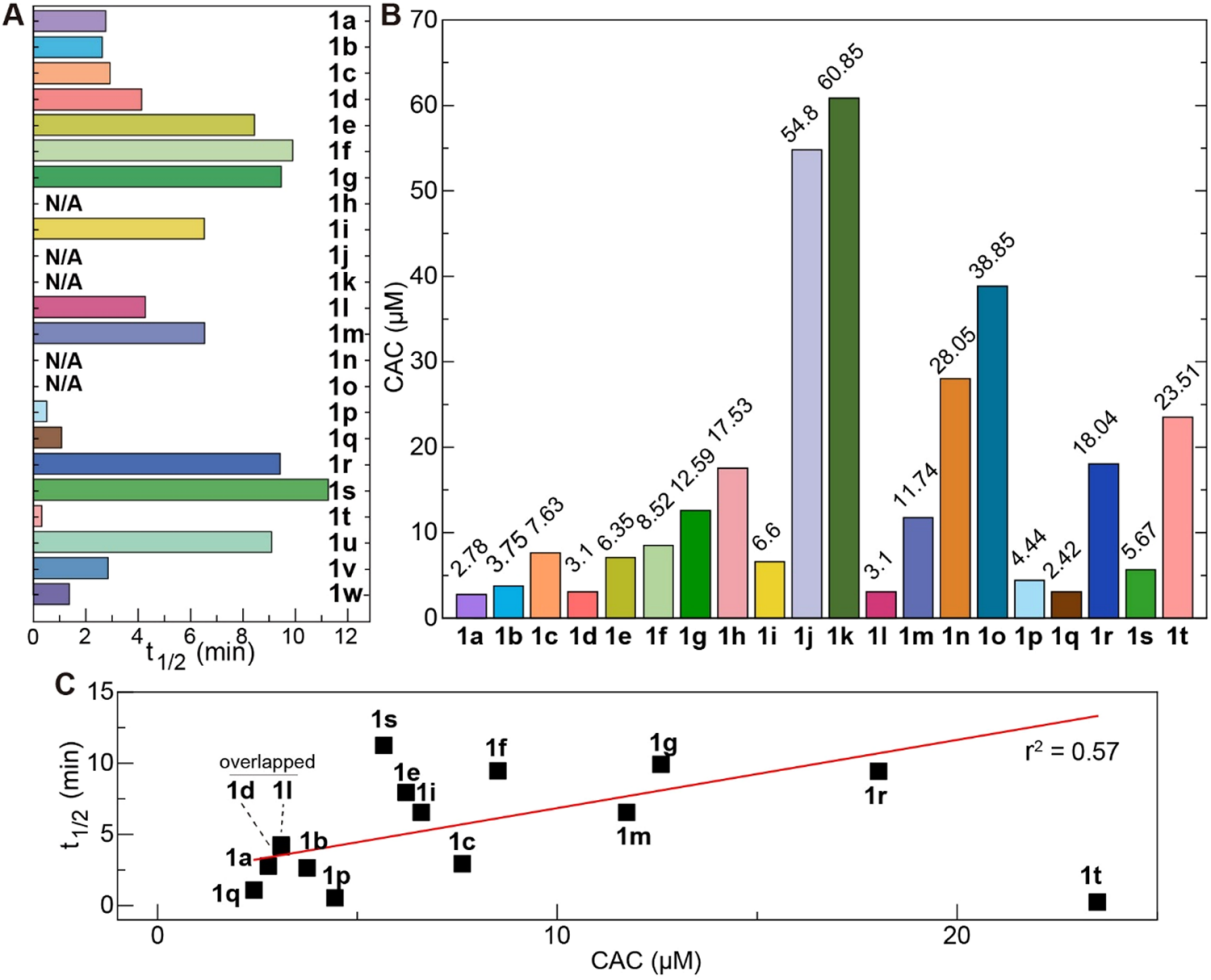
(A) *t*_1/2_ of CyMA-i precursors with decent Golgi targeting ability at 10 μM on HeLa cells. (B) CAC of CyMA-i precursors in MEM buffer determined by pyrene assay. (C) Linear correlation between kinetic trapping half-times (*t*_1/2_) and thermodynamic assembly propensity (CAC).

### Thermodynamic Thresholds for Potent Golgi-Targeting

To determine whether the same structural trends persist at a lower dose, we evaluated the imaging series at 500 nM. Under these conditions, only **1a** retained a strong time-dependent increase in Golgi fluorescence, whereas the aliphatic analogues **1b**–**1d** showed substantially weaker responses (Figure 1C). This divergence indicates that efficient CyMA function depends on whether the activated species can cross the threshold required for assembly under more dilute intracellular conditions.

The superior performance of **1a** at nanomolar concentration likely arises from the strong aromatic interactions provided by the d-Phe-d-Phe backbone,^39^ which enable productive assembly at a lower effective concentration than the aliphatic analogues. In this context, the d-Phe-d-Phe motif appears to provide the most favorable balance of hydrophobicity and packing interactions for stable Golgi accumulation. These data identify a thermodynamic requirement for high-potency targeting and explain why only a subset of the hydrophobic analogues remain effective at lower concentrations.

### Correlation between CAC and Kinetic Trapping

To quantify the role of self-assembly in CyMA performance, we compared the kinetic half-times of Golgi accumulation (*t*_1/2_) with the critical aggregation concentration (CAC) of the precursors measured in minimum essential medium (MEM) without phenol red (Figure 2).

The CAC values reveal strong sensitivity to backbone structure (Figure 2B). The parent scaffold **1a** exhibits a low CAC of 2.78 μM, consistent with efficient assembly driven by aromatic–aromatic interactions. The heterochiral analogues **1p** and **1q** also retained low CAC values of 4.44 and 2.42 μM, respectively, indicating that backbone stereochemistry has relatively little effect on assembly propensity within this scaffold. In contrast, the aliphatic analogues **1b**, **1c**, and **1d** showed weaker assembly, and the charged or highly polar analogues **1j**, **1k**, and **1t** exhibited substantially higher CAC values, consistent with their poorer cellular performance.

The kinetic data support this conclusion. Analogues with relatively low CAC values generally accumulated rapidly at the Golgi, whereas compounds with poor assembly propensity showed slow or undetectable targeting (Figure 2A). In particular, precursors such as **1h**, **1j**, **1k**, **1n**, and **1o** failed to establish a detectable Golgi-localized population (N/A in Figure 2A), despite containing the thioester trigger, suggesting that enzymatic activation alone is insufficient unless the resulting palmitoylated species can assemble before diffusing away.

Plotting *t*_1/2_ against CAC for the successful targeting analogues (**1a**, **1b**, **1c**, **1d**, **1e**, **1f**, **1g**, **1i**, **1l**, **1m**, **1p**, **1q**, **1r**, **1s**, except **1t**) gave a moderate positive correlation (*r* ^2^ = 0.57, Figure 2C), indicating that lower CAC generally favors faster trapping. The truncated control **1t** was excluded from the regression analysis due to its distinct, backbone-independent mode of cellular entry.^47^ This trend is consistent with the behavior of **1e**, which isolates side-chain bulk while maintaining a constant backbone length. Due to the steric bulk of its aromatic indole rings, **1e** exhibits an increased assembly threshold (CAC = 6.35 μM) and a slower accumulation rate (*t*_1/2_ ∼ 8 min), tracking closely along the regression line. This correlation suggests that assembly thermodynamics determine how rapidly accumulation proceeds once the assembly threshold is met. Compounds such as **1q** and **1w**, which trap rapidly despite modest differences in structure, illustrate that once sufficient assembly propensity is established, other kinetic factors become increasingly important.

The outliers in this analysis are also informative (Figure 2C). The tetrapeptide **1r** shows slower trapping than expected from its assembly propensity, suggesting that its increased length or steric bulk may hinder enzyme access or intracellular trafficking.^54–56^ By contrast, the truncated control **1t** exhibits unusually rapid apparent Golgi arrival (*t*_1/2_ ∼ 0.5 min) only at 10 μM, consistent with its weak assembly propensity and fast cellular entry by passive diffusion.^47^ Ultimately, this analysis demonstrates that cycling-and-trapping efficiency depends on the interplay between the thermodynamic propensity for assembly and the enzymatic rate of generating the building blocks for assembly. Together, these results clarify the distinct features of our system: while our earlier study uncovered the biological enzyme cascade behind the platform,^16^ the present quantitative correlation directly points to self-assembling ability as an important physicochemical parameter that, operating in conjunction with intracellular processing kinetics, allows transient enzyme-driven cycling to be converted into stable organelle-specific accumulation.

### Optimization of NTG for Golgi-Targeted Cytotoxicity

Having established the structural requirements for efficient Golgi targeting in the imaging series, we next examined whether modification of the NTG could convert the CyMA scaffold into a more potent Golgi-disrupting agent. To this end, the NBD of **1a** was replaced with a series of aromatic NTGs to generate **3a**–**3r** while retaining the d-Phe-d-Phe backbone and thioester trigger. Cell viability assays showed that **3a**, which contains a linear para-biphenyl motif, was the most potent analogue in the series, with a GI_50_ of ∼550 nM against HeLa cells (Figure 3A,B). HeLa cells were utilized as a well-characterized model for studying Golgi dynamics^57^ to validate this physicochemical SAR model, while normal cell lines were omitted to focus strictly on defining baseline chemical rules. Although these growth inhibition metrics evaluate global viability, both the inherent tumor-selectivity of the CyMA platform and the direct causal relationship between localized supramolecular assembly, structural Golgi disruption, and downstream cytotoxicity were extensively established in our previous study.^16^ This result indicates that the CyMA scaffold can be tuned from an imaging probe to a disruptive agent by modifying the NTG while preserving the same targeting mechanism.

**Figure 3:**
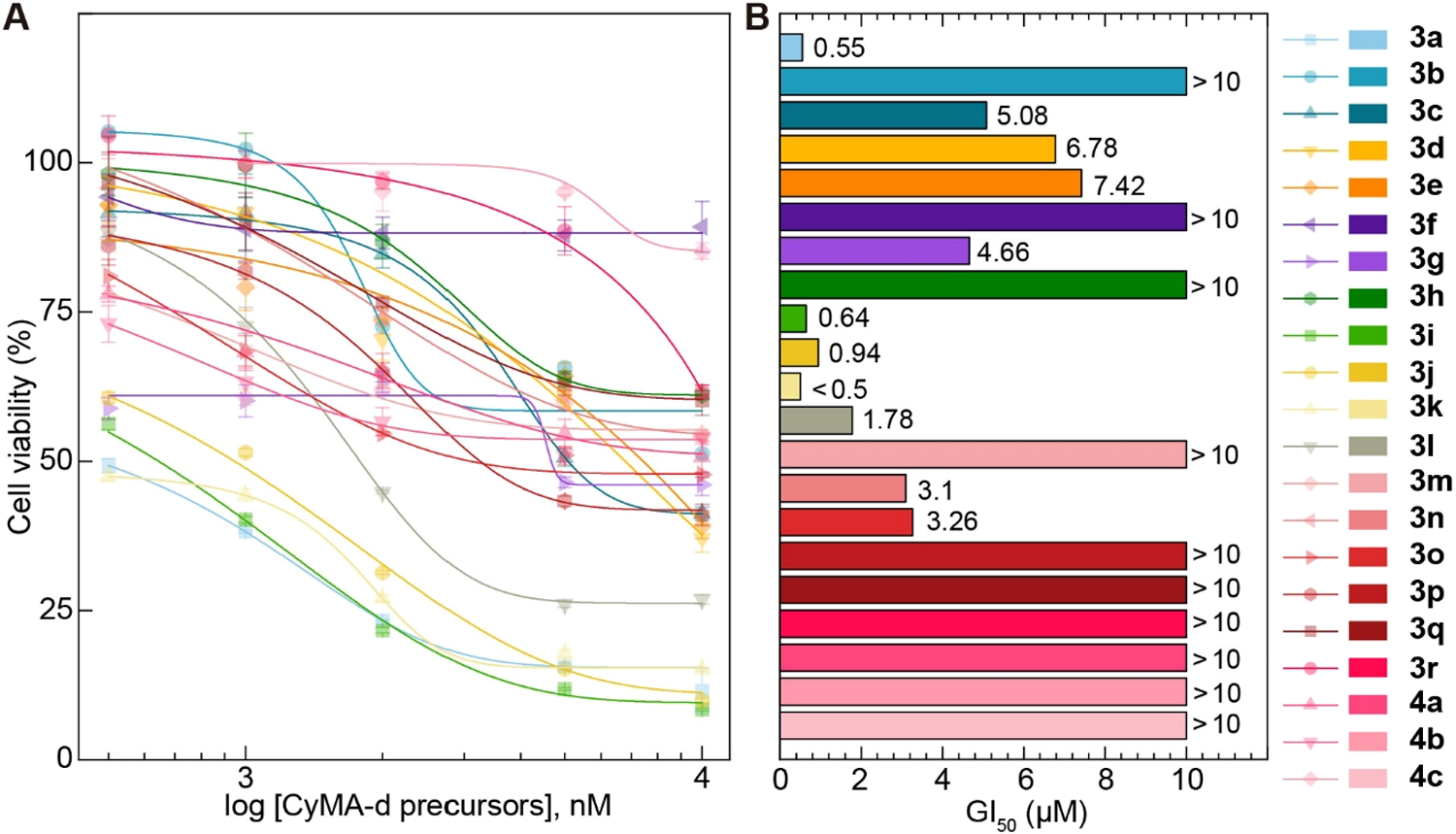
(A) Cell viability of HeLa cells treated with various CyMA-d precursors (**3a**–**4c**) for 24 h. (B) GI_50_ of **3a**–**4c** against HeLa cells for 24 h.

The SAR across the NTG series suggests that the molecular shape is a major determinant of cytotoxicity. Changing the biphenyl connectivity from para- to meta- or ortho, as in **3b** and **3c**, markedly reduced potency. Introducing flexible methylene linkers, as in **3d** and **3e**, was similarly detrimental, as were bulkier or differently shaped aromatic groups such as triphenylmethyl (**3f**), naphthoyl (**3q**), and phenyl (**3r**). These trends indicate that the para-biphenyl motif provides favorable geometry for productive packing within the Golgi-localized assemblies, whereas deviations from this linear architecture weaken the disruptive output.

In contrast, the series was more tolerant of electronic substitution than of major changes in the shape. Para-substituted biphenyl analogues bearing chloro (**3i**), methyl (**3j**), trifluoromethyl (**3k**), or cyano (**3l**) groups retained submicromolar to low micromolar potency. This behavior suggests that electronic tuning of the aromatic ring is less important than preserving a scaffold geometry compatible with dense local assembly. More hydrophilic substituents, such as carboxyl (**3g**) and acetate (**3h**), and heteroaromatic analogues **3m**–**3p** showed weaker activity, likely because greater polarity reduces the effective hydrophobic driving force for in situ assembly.

### Inhibitory Activity Depends on Self-Assembly Ability

To test directly whether the cytotoxicity of the CyMA-d series depends on the same assembly-driving backbone identified in the imaging series, we prepared analogues **4a**–**4c** in which the para-biphenyl NTG was retained, but the d-Phe-d-Phe sequence was replaced with weaker self-assembly motifs. All three analogues showed a pronounced loss of potency, with GI_50_ values greater than 10 μM (Figure 3B). These results demonstrate that the NTG alone is insufficient to confer activity. Instead, potent Golgi disruption requires the localized, high-density accumulation enabled by the d-Phe-d-Phe scaffold. This finding unifies the imaging and disruption series under a common design rule. In both contexts, the peptide backbone determines whether enzymatic cycling can generate a stable, nondiffusive population at the Golgi. Once that requirement is satisfied, the NTG determines the functional outcome, such as fluorescence reporting or cytotoxic disruption. Thus, biological activity in the CyMA system is controlled not simply by the identity of the appended group, but by how effectively that group is presented within a Golgi-localized supramolecular assembly.^16^

## Conclusions

In this work, we defined the key structural features that govern the activity of CyMA for Golgi targeting and disruption. By systematically varying the peptide backbone, thioester warhead, and N-terminal capping group, we established that the self-assembling ability is the primary determinant of CyMA performance. In particular, the d-Phe-d-Phe backbone provided the most favorable assembly propensity and enabled efficient accumulation even at nanomolar concentrations, whereas analogues with a weaker assembly propensity showed reduced localization and diminished biological activity. This observation suggests that fast cell entry and rapid accumulation are essential for the potency of organelle-targeting drugs.

Our results also show that different structural elements make distinct contributions to CyMA function. The peptide backbone strongly influences whether enzymatic cycling can produce a stable, nondiffusive Golgi-localized population,^16^ while the thioester warhead, cellular uptake, and intracellular trafficking modulate the kinetics and extent of accumulation. The thioester warhead primarily modulates the kinetics of intracellular activation, and the N-terminal capping group tunes the functional output of the resulting assemblies, including Golgi-disrupting cytotoxicity. The CAC-*t*_1/2_ relationship further supports a model in which assembly thermodynamics sets the threshold for kinetic trapping, while enzymatic turnover and cellular uptake influence the rate of accumulation. Importantly, while these in vitro CAC values reflect the intact precursors rather than the active, transiently S-palmitoylated metabolites, they serve as a reliable proxy because our structural modifications dictate the foundational intermolecular forces driving the assembly.

Together, these findings establish a structure–activity framework for CyMA and provide design principles for developing enzyme-switched molecular assemblies for organelle-targeted drugs. More broadly, this work shows how coupling intracellular enzymatic cycling to supramolecular assembly can generate stable subcellular localization without relying on conventional ligand–receptor recognition. Such a strategy may offer a useful foundation for the future development of organelle-directed molecular probes and perturbation agents.

In addition, future studies incorporating non-natural amino acids, such as p-phenyl-phenylalanine (Bip), which displays a stronger self-assembly propensity than phenylalanine,^58,59^ may further expand the available design space. Taken together, these results suggest the possibility of a new class of dynamic supramolecular medicines that harness endogenous metabolic pathways for precision therapy^60^ while also pointing to a broader shift in molecular design from targeting individual proteins toward modulating organelle-level biological processes. Moreover, because palmitate availability is elevated in metastatic and premetastatic niches, including increased palmitate in lung interstitial fluid from patients with breast cancer,^61^ palmitoylation-dependent CyMA is particularly attractive for preferential Golgi targeting in the tumor microenvironment, although this context-dependent selectivity remains to be carefully designed and tested.

## Experimental Section

### General Information

2-Cl-trityl chloride resin (1.0 mmol/g), HBTU, and Fmoc-protected amino acids were obtained from GL Biochem (Shanghai, China). Solvents, 2-mercaptoethanol, and *N*,*N*-diisopropylethylamine (DIEA) were purchased from Fisher Scientific. Alfa Aesar provided the 4-chloro-7-nitrobenzofurazan, while the Indofine Chemical Company was the source for β-alanine. The reagents acetyl chloride, acetoxyacetyl chloride, and 2-naphthoic acid were acquired from TCI America. Various biphenyl and pyridine derivatives, including biphenyl-3-carboxylic acid, [1,1′-biphenyl]-2-carboxylic acid, 4-biphenylacetic acid, 4-(4-pyridyl)­benzoic acid, 4-pyridin-3-yl-benzoic acid, 4-(2-pyridyl)­benzoic acid, and 2,2′-bipyridine-5-carboxylic acid, were sourced from 1PlusChem. Enamine was the supplier for 4-benzylbenzoic acid. AmBeed provided both methyl 3-chloro-3-oxopropanoate and 4′-hydroxy-[1,1′-biphenyl]-4-carboxylic acid. Reagents and solvents from commercial vendors were utilized without further purification. Cell culture materials, including penicillin–streptomycin (PS), fetal bovine serum (FBS), and minimum essential medium (MEM), were obtained from Gibco.

### Characterization and Purification

An Agilent 1100 Series system with an XTerra C18 RP column was used for the reversed-phase HPLC purification of all precursors and final compounds. The mobile phases consisted of HPLC-grade water and acetonitrile, both containing 0.1% TFA. A Bruker Elute PLUS UHPLC system coupled with a Bruker timsTOF Pro instrument was used to acquire LC-MS spectra. A ZEISS LSM 880 confocal laser scanning microscope was employed for the fluorescence imaging.

### Synthesis of NBD-β-alanine

As shown in Scheme S1, a MeOH solution (60 mL) containing NBD-Cl (1 g, 5 mmol) was introduced dropwise into a stirred 10 mL aqueous solution of β-alanine (490 mg, 5.5 mmol) and potassium carbonate (2.07 g, 16.5 mmol). The reaction was maintained at ambient temperature for 6 h under a nitrogen atmosphere. Upon completion, the methanol was stripped away using a rotary evaporator, and the remaining solution was adjusted to pH 3 with 1 N HCl. This aqueous phase was then subjected to extraction with diethyl ether. The collected organic fractions were dried over anhydrous sodium sulfate and then concentrated under reduced pressure. The final product, obtained as a dark-yellow powder, was utilized directly in solid-phase peptide synthesis (SPPS).

### Synthesis of CyMA Precursors (1a–1w, 3a–3r, 4a–4c)

The synthesis of CyMA precursors followed standard Fmoc-based solid-phase peptide synthesis (SPPS) protocols on 2-chlorotrityl chloride resin, utilizing Fmoc amino acids with specific side-chain protection.^16^ One gram of resin was swollen in anhydrous dichloromethane (DCM) for 30 min. To install the thiol linker, the resin was incubated overnight at room temperature with 10 equiv of 2-mercaptoethanol dissolved in a 1:1 (v/v) mixture of DCM/DMF. Selective attachment to the resin support was driven by the high nucleophilicity of the mercaptoethanol thiol group. The resin was then thoroughly washed with DMF to eliminate the excess reagents.

Subsequently, the first Fmoc-protected amino acid (1.5 equiv) was coupled to the linker-modified resin using DCC (2 equiv) and catalytic DMAP in DMF, with overnight incubation. Deprotection of the Fmoc group was performed using 20% piperidine in DMF, and chain elongation continued through sequential HBTU-mediated couplings. After the peptide sequence was completed, the N-terminus was capped on resin with various functional groups (NTGs) to generate the different CyMA analogues. Cleavage from the resin was achieved by applying a TFA cocktail (95% TFA, 2.5% TIPS, and 2.5% H_2_O) for 1 h. The released thiopeptides were dissolved in anhydrous trifluoroacetic acid (TFA) and cooled in an ice–water bath under a nitrogen atmosphere. Subsequently, four equivalents of the designated acyl chloride were introduced dropwise into the TFA solution, and the reaction was allowed to reach ambient temperature. Following a 3 h stirring period, the reaction was terminated by quenching with ice-cold water. Volatiles were removed under reduced pressure, and the resulting crude products were purified via reversed-phase HPLC (Scheme S2) to yield final compounds with >95% purity as confirmed by HPLC analysis.

## Supplementary Material





## Notes

Additional experimental details, materials, and methods (PDF)
Molecular formula strings for all newly synthesized compounds (CSV)

## Abbreviations Used

CAC: critical aggregation concentration
CLSM: confocal laser scanning microscopy
CyMA: cycling molecular assemblies
EISA: enzyme-instructed self-assembly
NBD: nitrobenzoxadiazole
NTG: *N*-terminal capping group
RP-HPLC: reverse-phase high-performance liquid chromatography
SAR: structure–activity relationship
TE: thioesterase
zDHHC: palmitoyl acyltransferase
