# Supplementary material for: Structure–Activity Relationship of Cycling Molecular Assemblies for Golgi Targeting and Disruption
Source: J Med Chem. 2026 Jul 8;69(14):17310–8. doi: 10.1021/acs.jmedchem.6c01269 (PMC13403230; doi:10.1021/acs.jmedchem.6c01269)
Supplement: Supplementary file 1 [file jm6c01269_si_001.pdf]

Supplementary Materials for

Structure-Activity Relationship of Cycling Molecular Assemblies for Golgi Targeting and Disruption

Weiyi Tan,[1] Qiuxin Zhang,[1] Pengyu Hong,[2] Bing Xu[1,*]

1. Department of Chemistry, Brandeis University, Waltham, MA 02453, United States
2. Department of Computer Science, Brandeis University, Waltham, MA 02453, United States

*Correspondence: bxu@brandeis.edu

Table of Contents

- **Materials and Methods** .... Page S4
  - Materials .... Page S4
  - Instruments .... Page S4
- **Experimental Procedures** .... Page S4
  - Synthesis of NBD-β-Alanine........ Page S4
  - CyMA precursors synthesis........ Page S5
  - Critical Aggregation Concentration (CAC) Determination........ Page S5
  - Cell Culture........ Page S5
  - Confocal microscopy .... Page S6
  - Quantification of fluorescence intensity at Golgi........ Page S6
  - Cell Viability (MTT) assay........ Page S7
- **Supplementary Figures** .... Page S8
  - **Figure S1.** Dual-Channel Colocalization Analysis of **1a** with GALNT2-RFP Marker .... Page S8
  - **Figure S2.** CLSM images of HeLa cells treated with **1a** (10 μM) over 24 minutes. .... Page S8
  - **Figure S3.** CLSM images of HeLa cells treated with **1b** (10 μM) over 24 minutes. .... Page S9
  - **Figure S4.** CLSM images of HeLa cells treated with **1c** (10 μM) over 24 minutes. .... Page S9
  - **Figure S5.** CLSM images of HeLa cells treated with **1d** (10 μM) over 24 minutes. .... Page S10
  - **Figure S6.** CLSM images of HeLa cells treated with **1e** (10 μM) over 24 minutes. .... Page S10
  - **Figure S7.** CLSM images of HeLa cells treated with **1f** (10 μM) over 24 minutes. .... Page S11
  - **Figure S8.** CLSM images of HeLa cells treated with **1g** (10 μM) over 24 minutes. .... Page S11
  - **Figure S9.** CLSM images of HeLa cells treated with **1h** (10 μM) over 24 minutes. .... Page S12
  - **Figure S10.** CLSM images of HeLa cells treated with **1j** (10 μM) over 24 minutes. .... Page S12
  - **Figure S11.** CLSM images of HeLa cells treated with **1k** (10 μM) over 24 minutes. .... Page S13

- **Figure S12.** Cell viability of HeLa cells treated with **1h**, **1j**, **1k**, **1n**, **1o** for 24 hours .... Page S13
- **Figure S13.** CLSM images of HeLa cells treated with **1r** (10 μM) over 24 minutes. .... Page S14
- **Figure S14.** CLSM images of HeLa cells treated with **1s** (10 μM) over 24 minutes. .... Page S14
- **Figure S15.** CLSM images of HeLa cells treated with **1t** (10 μM) over 24 minutes. .... Page S15
- **Figure S16.** CLSM images of HeLa cells treated with **1p** (10 μM) over 24 minutes. .... Page S15
- **Figure S17.** CLSM images of HeLa cells treated with **1q** (10 μM) over 24 minutes. .... Page S16
- **Figure S18.** CLSM images of HeLa cells treated with **1v** (10 μM) over 24 minutes. .... Page S16
- **Figure S19.** CLSM images of HeLa cells treated with **1w** (10 μM) over 24 minutes. .... Page S17
- **Figure S20.** CLSM images of HeLa cells treated with **1u** (10 μM) over 24 minutes. .... Page S17
- **Figure S21.** LC-HRMS of **1a–1i** .... Page S18
- **Figure S22.** LC-HRMS of **1j–1q** .... Page S19
- **Figure S23.** LC-HRMS of **1r–1w** .... Page S20
- **Figure S24.** LC-HRMS of **3a–3h** .... Page S21
- **Figure S25.** LC-HRMS of **3i–3p** .... Page S22
- **Figure S26.** LC-HRMS of **3q–3r**, **4a–4c** .... Page S23

References .... Page S23

Materials and Methods

Materials

2-Cl-trityl chloride resin (1.0 mmol/g), HBTU, and Fmoc protected amino acid were obtained from GL Biochem (Shanghai, China). Solvents, 2-mercaptoethanol, and N,N-diisopropylethylamine (DIEA) were purchased from Fisher Scientific. Alfa Aesar provided the 4-chloro-7-nitrobenzofurazan, while Indofine Chemical Company was the source for β-alanine. The reagents acetyl chloride, acetoxyacetyl chloride, and 2-naphthoic acid were acquired from TCI America. Various biphenyl and pyridine derivatives, including biphenyl-3-carboxylic acid, [1,1'-biphenyl]-2-carboxylic acid, 4-biphenylacetic acid, 4-(4-pyridyl)benzoic acid, 4-pyridin-3-yl-benzoic acid, 4-(2-pyridyl)benzoic acid, and 2,2'-bipyridine-5-carboxylic acid, were sourced from 1PlusChem. Enamine was the supplier for 4-benzylbenzoic acid. AmBeed provided both methyl 3-chloro-3-oxopropanoate and 4'-hydroxy-[1,1'-biphenyl]-4-carboxylic acid. Reagents and solvents from commercial vendors were utilized without further purification. Cell culture materials, including penicillin-streptomycin (PS), fetal bovine serum (FBS), and Minimum Essential Medium (MEM), were obtained from Gibco.

Instruments

An Agilent 1100 Series system with an XTerra C18 RP column was used for the reverse-phase HPLC purification of all precursors and final compounds. The mobile phases consisted of HPLC-grade water and acetonitrile, both containing 0.1% TFA. A Bruker Elute PLUS UHPLC system coupled with a Bruker timsTOF Pro was used to acquire LC-MS spectra. Peak area integration confirmed that all tested compounds possess a chemical purity of >90%. For 1k, 1n, and 1w, the two close peaks sharing the exact same m/z value represent the same compound existing in different self-aggregated states under the chromatographic conditions.[1-2] A ZEISS LSM 880 confocal laser scanning microscope was employed for fluorescence imaging.

Methods

Synthesis of NBD-β-Alanine

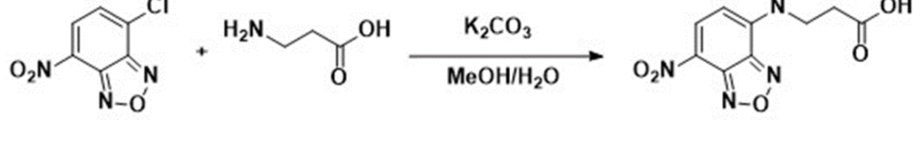

Scheme S1. Reaction scheme for the synthesis of NBD-β-Alanine.

CyMA precursors synthesis

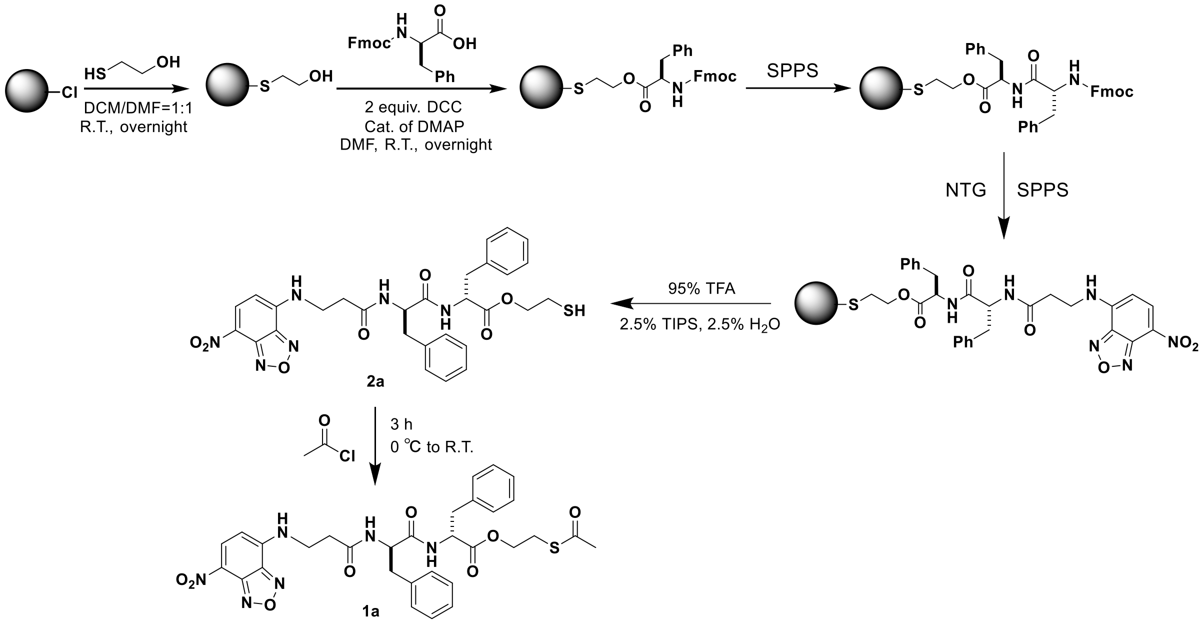

Scheme S2. Synthetic procedure of the representative CyMA precursor, **1a**.

CAC determination

To determine the critical aggregation concentration (CAC) of the CyMA precursors, a fluorescence assay was performed using a saturated solution of pyrene in Minimum Essential Medium (MEM, without phenol red) as the probing environment. Briefly, a saturated solution of pyrene was prepared in MEM buffer and utilized to formulate a concentration gradient of the CyMA variants. Fluorescence emission spectra were recorded using a microplate reader with an excitation wavelength of 335 nm, monitoring the emission between 360 nm and 500 nm. The ratio of the intensities of the first and third vibrational peaks (I_1 at ~378 nm and I_3 at ~393 nm) was calculated and plotted against the logarithmic concentration of the precursor. The CAC was identified as the concentration at the inflection point where the I_1/I_3 ratio began to decrease significantly, indicating the onset of assembly formation and the subsequent sequestration of pyrene into the hydrophobic domains of the nanostructures.[3]

Cell culture

HeLa cells were obtained from ATCC and authenticated via STR profiling (CellCheck 9 - human, IDEXX) to confirm a 100% identity match. The cells were maintained in Minimum Essential Medium (MEM) supplemented with 10% fetal bovine serum (FBS) and a 1% antibiotic-antimycotic solution (100 U/mL penicillin, 100 µg/mL streptomycin). Cultures were incubated at 37 °C in a humidified atmosphere containing 5% CO_2 at 37 °C.

Confocal microscopy

Imaging was performed using #1.5 glass-bottom confocal dishes (35 mm dish, 20 mm well). For standard live-cell observations, cells were seeded at a density of 1.0×10^5 per dish and allowed to adhere for 24 hours. The growth medium was replaced with fresh medium containing the target compound, and the samples were incubated at 37 °C in 5% CO_2 for the specified duration. Nuclei were counterstained with Hoechst 33342 for 10 minutes, followed by four 1-mL washes with Live Cell Imaging Solution to eliminate background fluorescence. For time-lapse CLSM studies, the same seeding protocol was followed, with cells washed three times before nuclear staining and four times after. Cell positioning and focal stability were managed using the 405 nm signal and the Nikon Perfect Focus System. Precursors were added to the dish in fresh imaging solution just before recording. Time-lapse datasets were captured at 1-minute intervals or in continuous mode, with multi-channel images archived for quantitative analysis.

Quantification of fluorescence intensity at Golgi

Automated image analysis was conducted to quantify single-cell responses, with pixel values normalized to a relative scale of [0, 1].

(a) Nuclear Segmentation. The Otsu method[4] was used on nucleus-staining frames to automatically determine an intensity threshold for detecting foreground pixels corresponding to nuclei. The minimum threshold was set to 0.6 out of 1.0. Morphological operations (open, fill, and erode) were applied to remove noise, fill the holes in the foreground segments, and separate foreground pixels into segments corresponding individual nuclei. Some nuclei near image boundaries were discarded.

(b) Signal Detection. Thresholds for the reaction signal were derived from the final video frame, set to exceed 99% of background pixels. Foreground segments smaller than 4 pixels were discarded. Each signal segment was assigned to the nearest nucleus to define cellular boundaries.

(c) Golgi Detection. To ensure unbiased kinetic quantification, the structural Golgi mask was generated by applying a uniform manual threshold of 0.05 exclusively to the independent GALNT2-RFP organelle marker channel. Because the fluorescence intensity of this transfected marker is stable and completely independent of the treated chemical variants, this uniform threshold establishes a consistent, structurally objective region of interest (ROI) across the entire series. The green fluorescence intensities of the various CyMA analogues were then measured over time strictly within this marker-defined mask, rendering the quantification completely independent of individual analogue brightness or spatial distribution patterns. This

mask enabled the classification of fluorescence signals as intra-Golgi or extra-Golgi for kinetic tracking.

Cell Viability (MTT) assay

The metabolic activity of HeLa cells following treatment was evaluated using the MTT assay. Cells were seeded in 96-well plates (1.0×10^5 cells/well) and incubated for 24 hours to ensure attachment. The media were then replaced with fresh growth medium containing varied concentrations of the compounds. After incubation for 24, 48, or 72 hours, 10 μL of MTT (5 mg/mL) was added to each well, followed by 4 hours of dark incubation at 37 °C. Formazan crystals were solubilized in 100 μL of 10% SDS-HCl, and the absorbance at 595 nm was measured via a microplate reader. Data are presented as mean ± standard deviation from three independent replicates. For 7-day toxicity assessments, the seeding density was reduced to 5,000 cells/well, with fresh compound-containing medium supplied every three days.

Supplementary figures

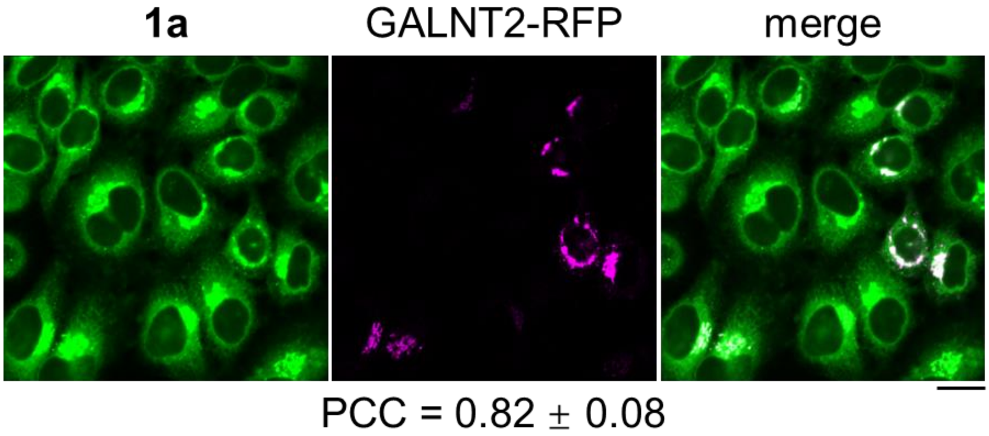

Figure S1. Colocalization analysis of **1a** (5 μM, 4 minutes) with Golgi resident protein, GALNT2, which was transfected with CellLight™ Golgi-RFP, BacMam 2.0. Scale bar = 20 μm.

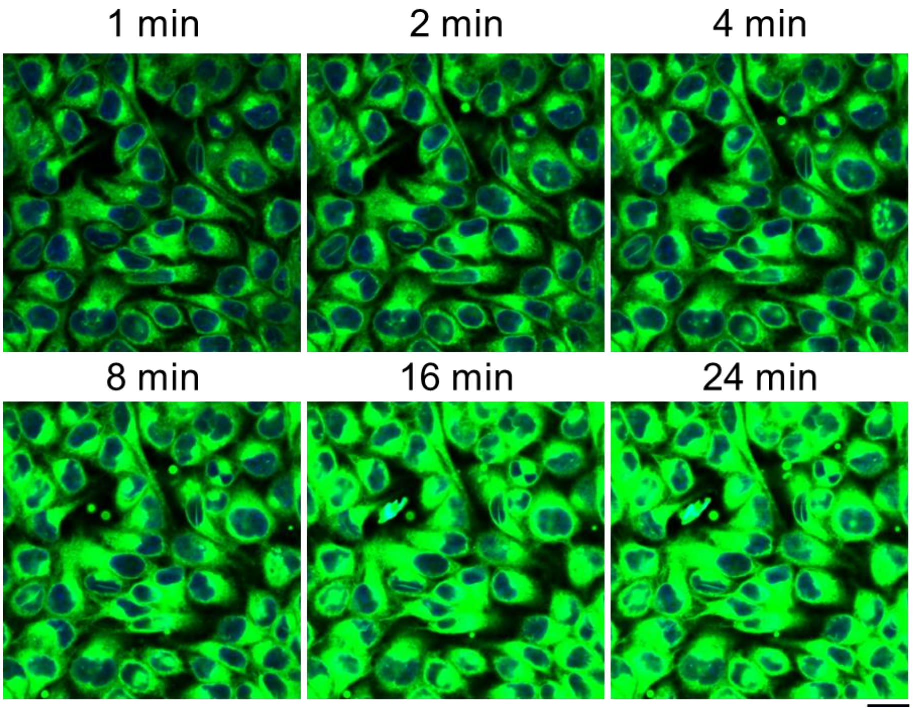

Figure S2. CLSM images of HeLa cells treated with **1a** (10 μM) over 24 minutes. Scale bar = 20 μm.

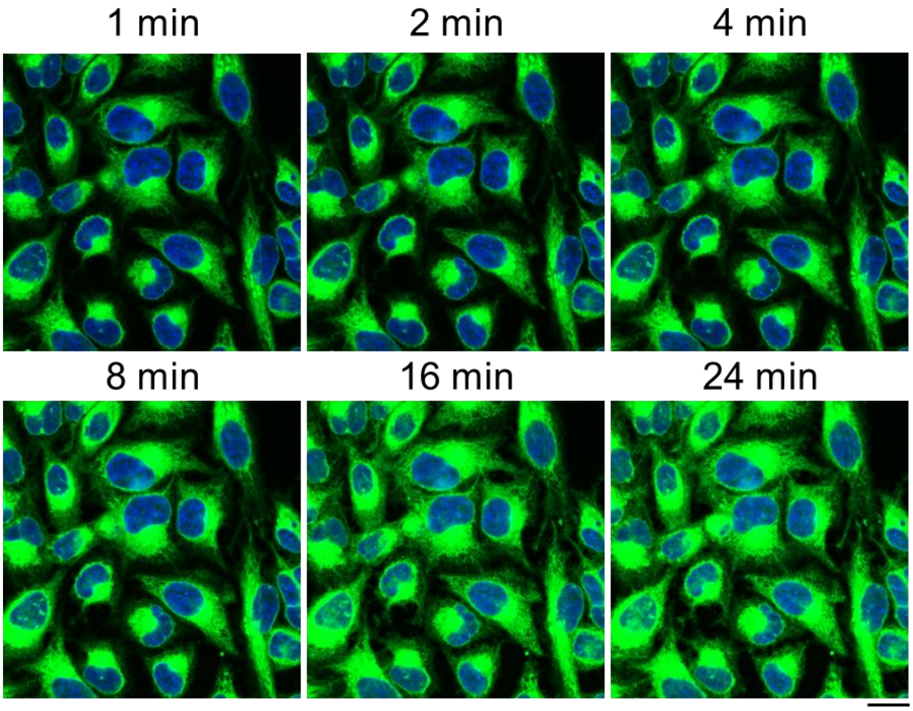

Figure S3. CLSM images of HeLa cells treated with **1b** (10 μM) over 24 minutes. Scale bar = 20 μm.

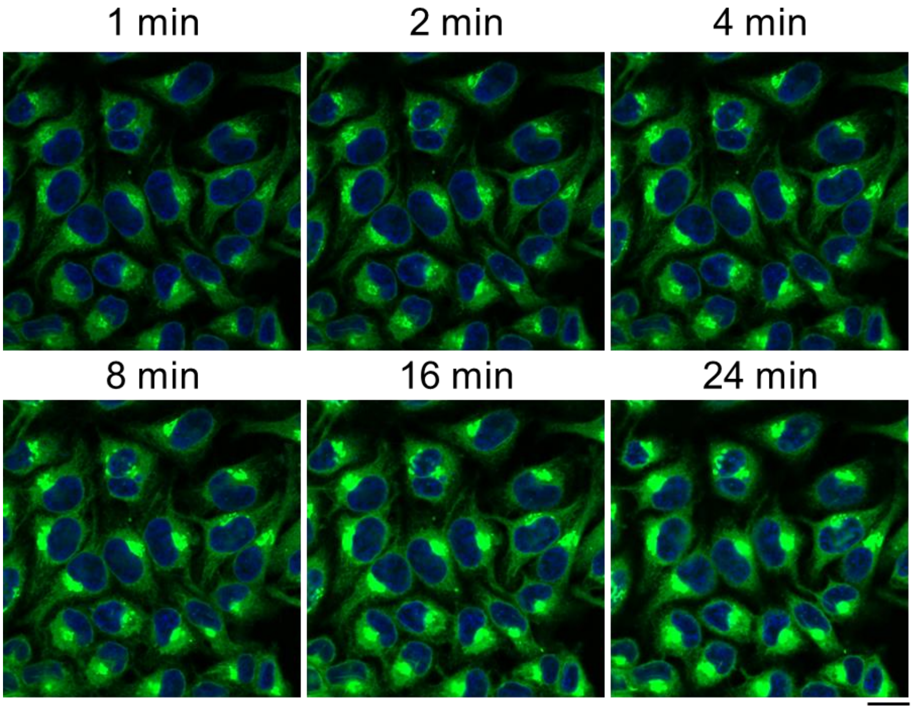

Figure S4. CLSM images of HeLa cells treated with **1c** (10 μM) over 24 minutes. Scale bar = 20 μm.

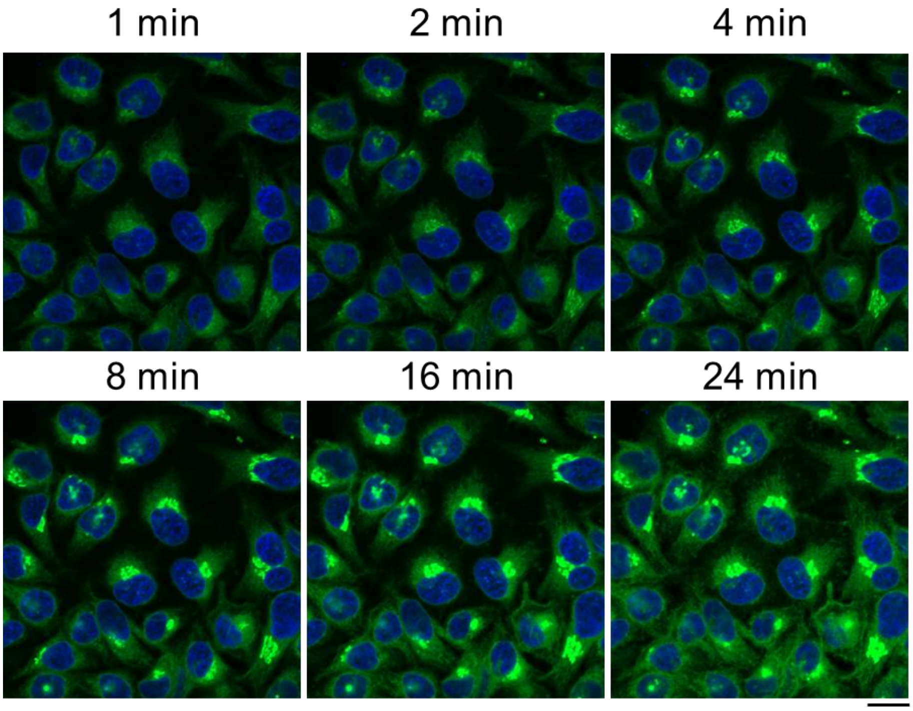

Figure S5. CLSM images of HeLa cells treated with **1d** (10 μM) over 24 minutes. Scale bar = 20 μm.

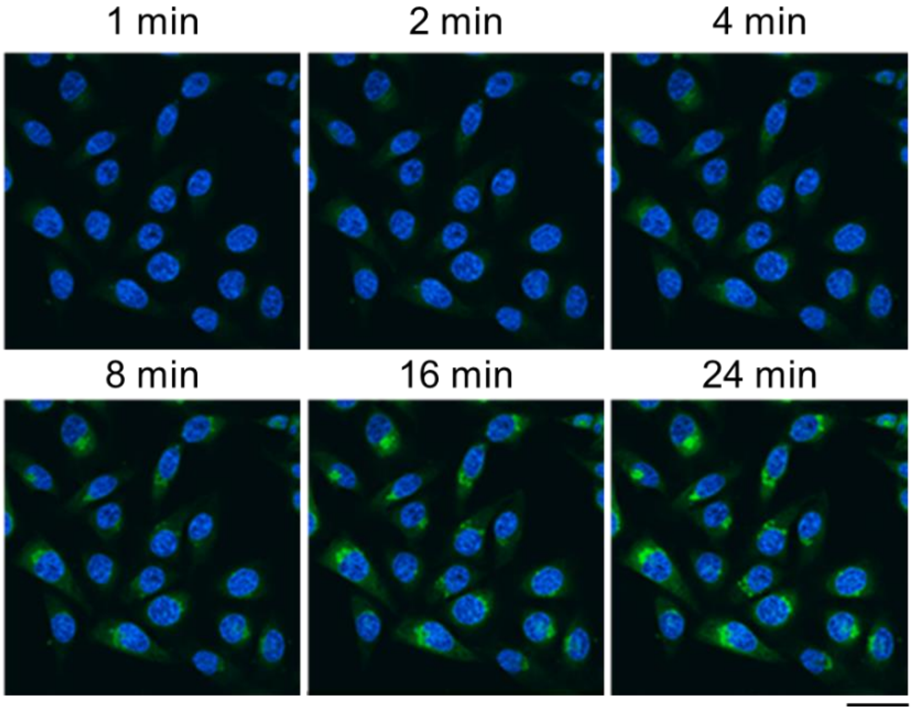

Figure S6. CLSM images of HeLa cells treated with **1e** (10 μM) over 24 minutes. Scale bar = 20 μm.

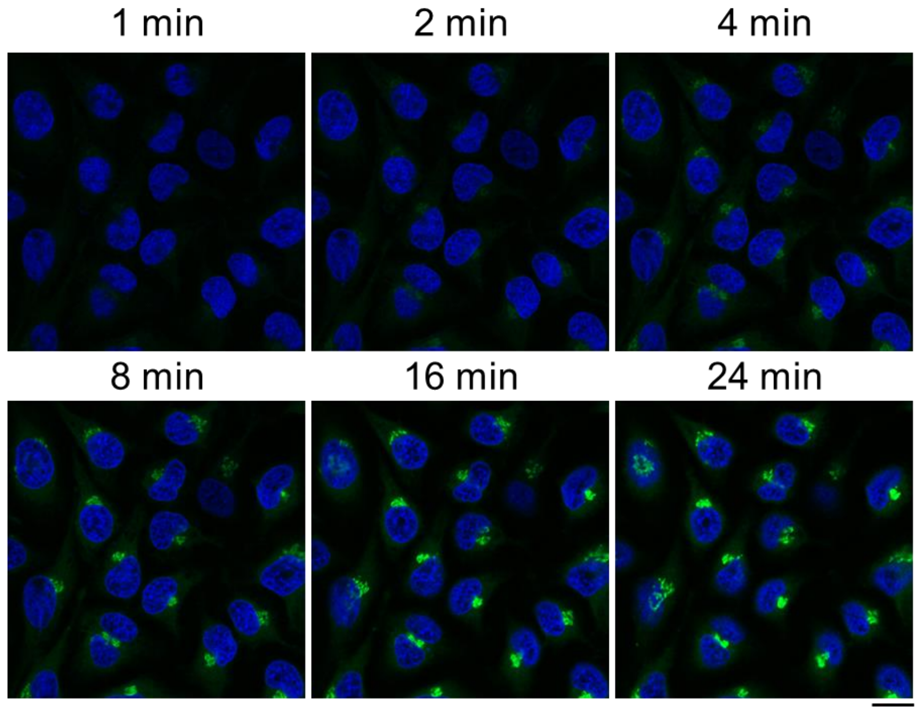

Figure S7. CLSM images of HeLa cells treated with **1f** (10 μM) over 24 minutes. Scale bar = 20 μm.

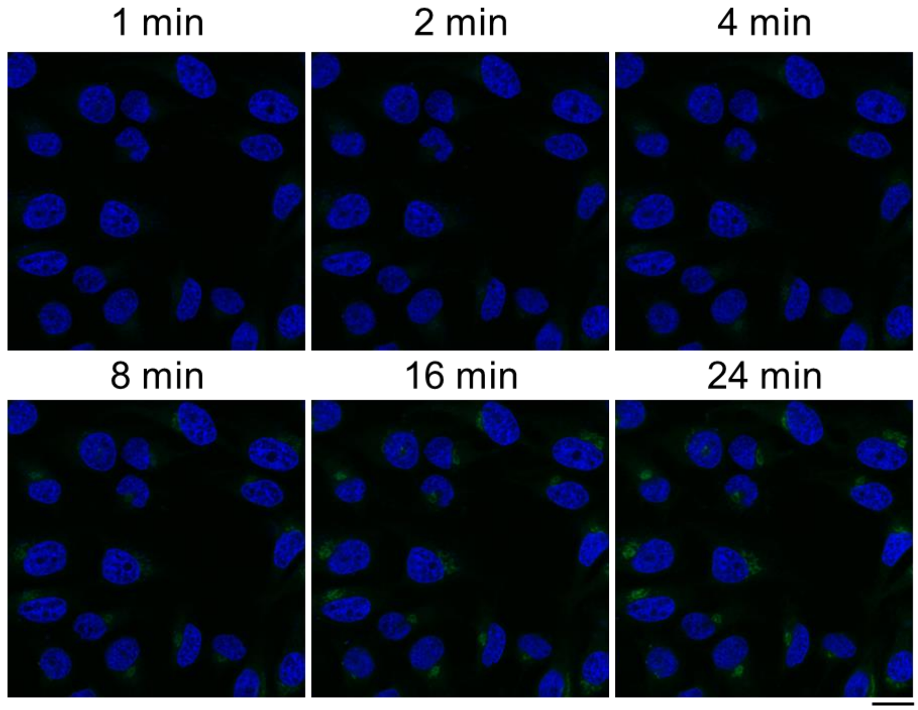

Figure S8. CLSM images of HeLa cells treated with **1g** (10 μM) over 24 minutes. Scale bar = 20 μm.

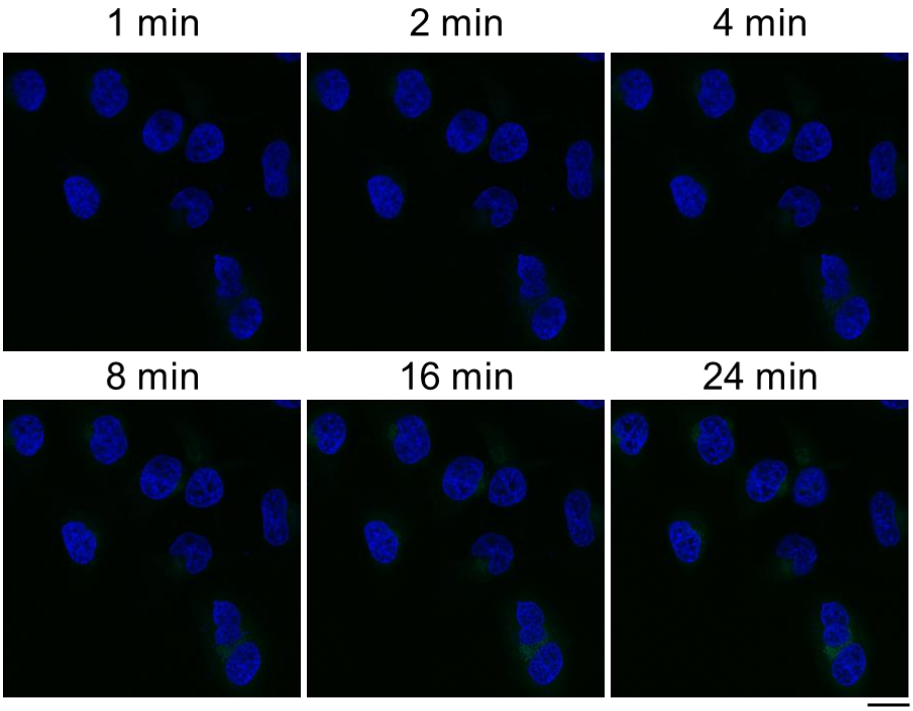

Figure S9. CLSM images of HeLa cells treated with **1h** (10 μM) over 24 minutes. Scale bar = 20 μm.

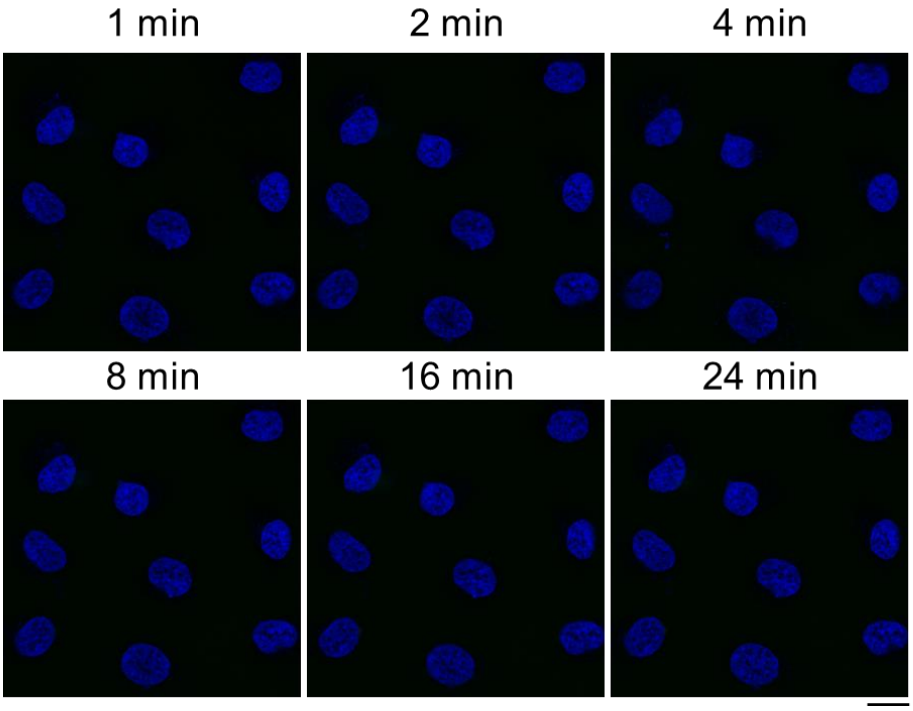

Figure S10. CLSM images of HeLa cells treated with **1j** (10 μM) over 24 minutes. Scale bar = 20 μm.

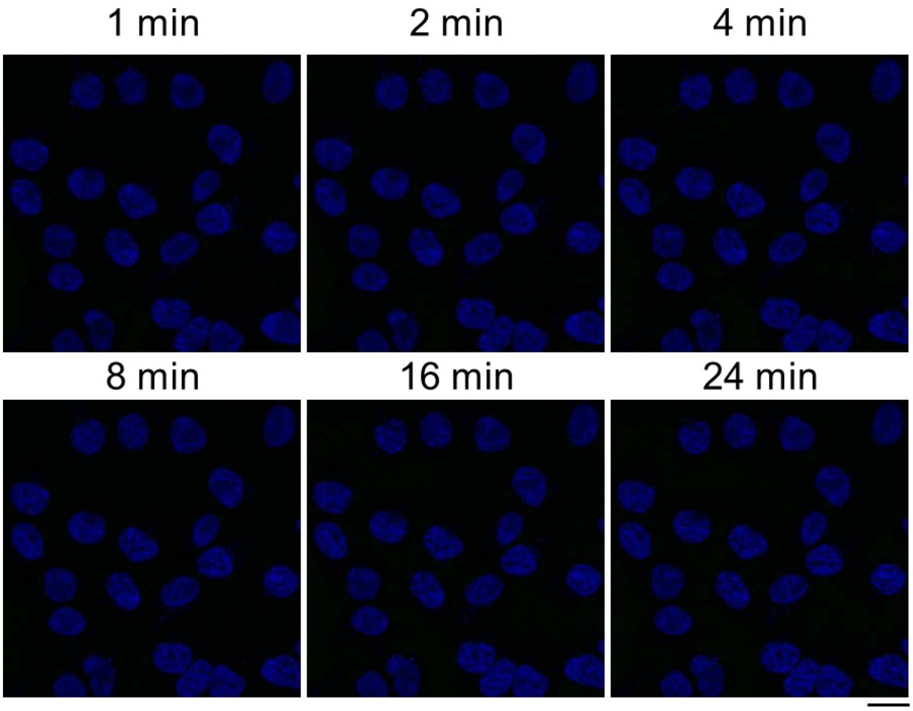

Figure S11. CLSM images of HeLa cells treated with **1k** (10 μM) over 24 minutes. Scale bar = 20 μm.

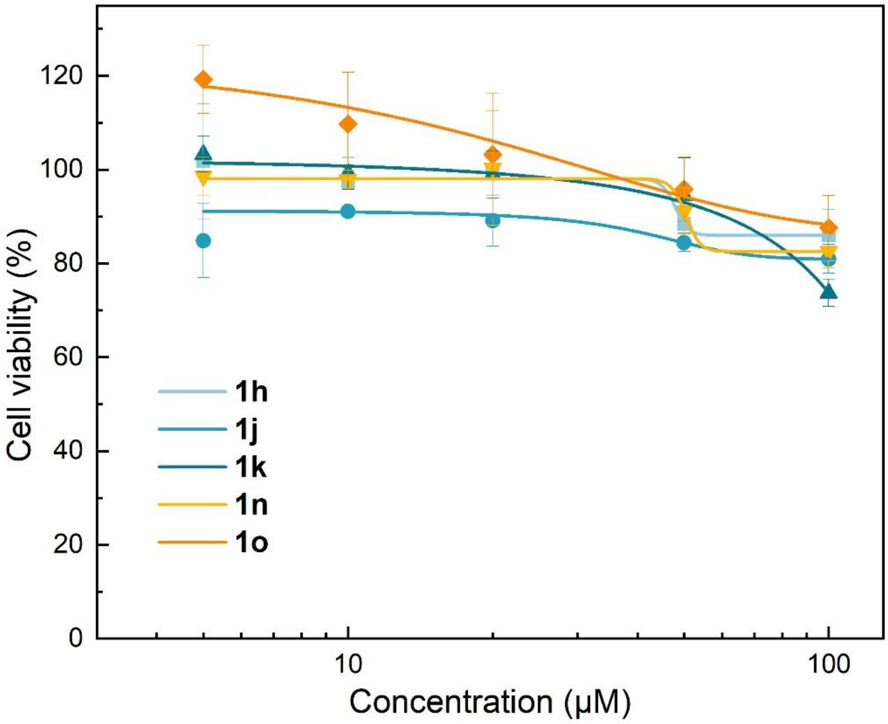

Figure S12. Cell viability of HeLa cells treated with **1h**, **1j**, **1k**, **1n**, **1o** for 24 hours.

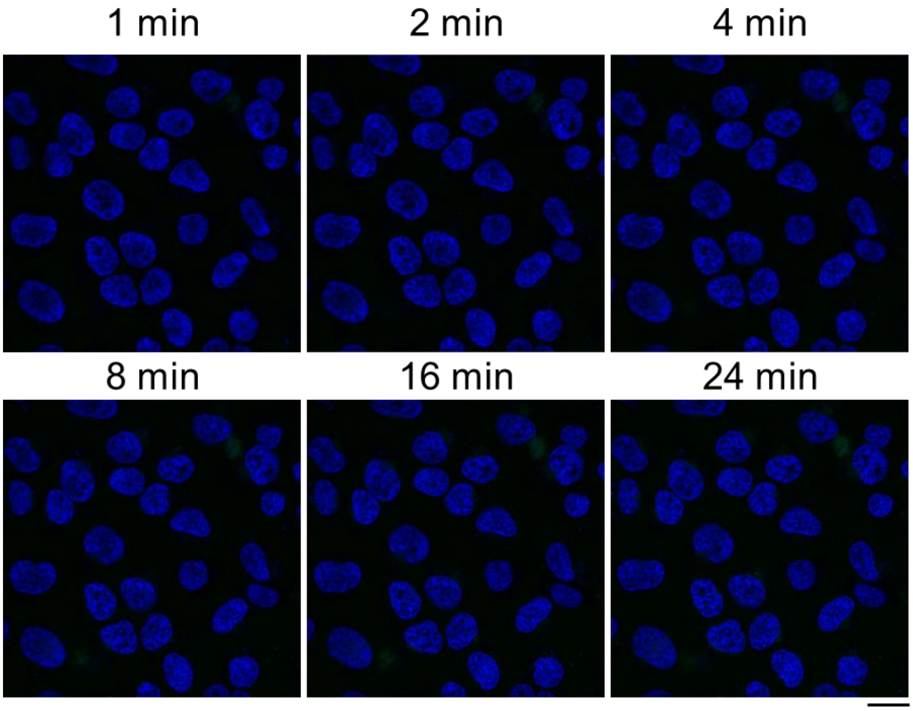

Figure S13. CLSM images of HeLa cells treated with **1r** (10 μM) over 24 minutes. Scale bar = 20 μm.

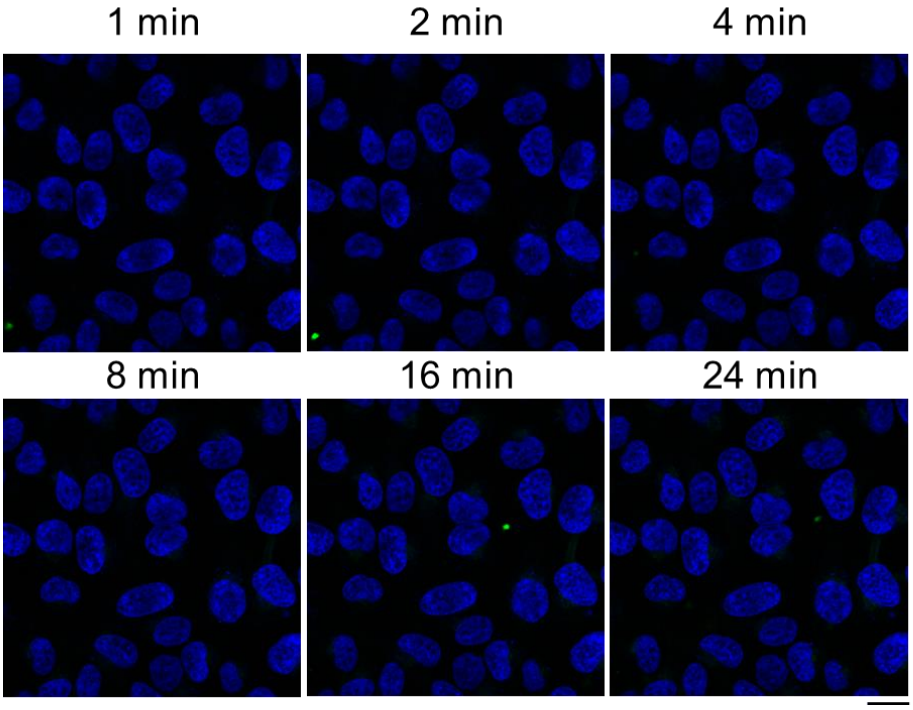

Figure S14. CLSM images of HeLa cells treated with **1s** (10 μM) over 24 minutes. Scale bar = 20 μm.

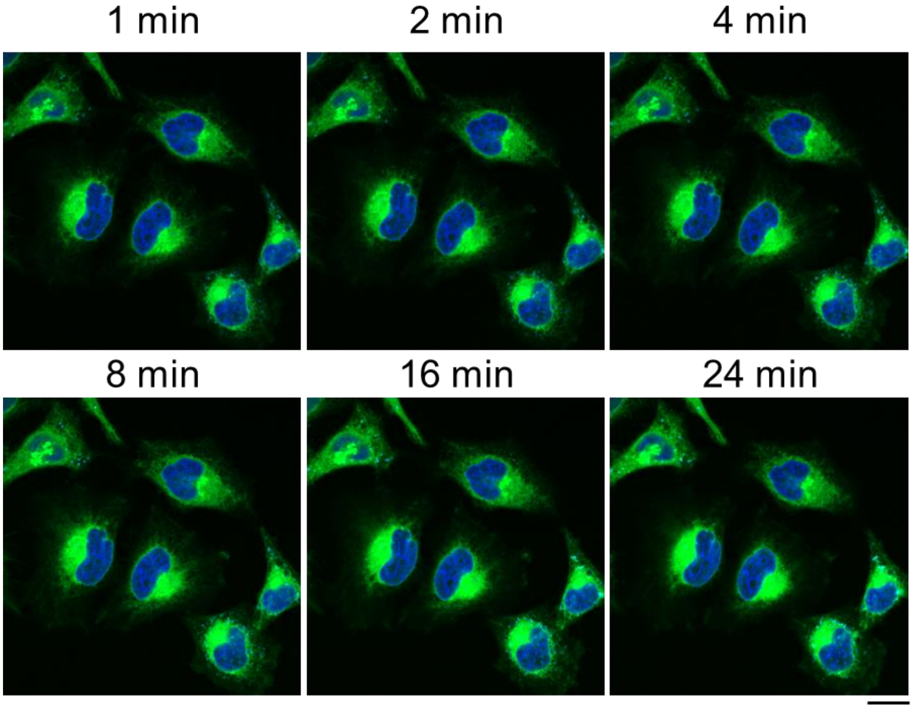

Figure S15. CLSM images of HeLa cells treated with **1t** (10 μM) over 24 minutes. Scale bar = 20 μm.

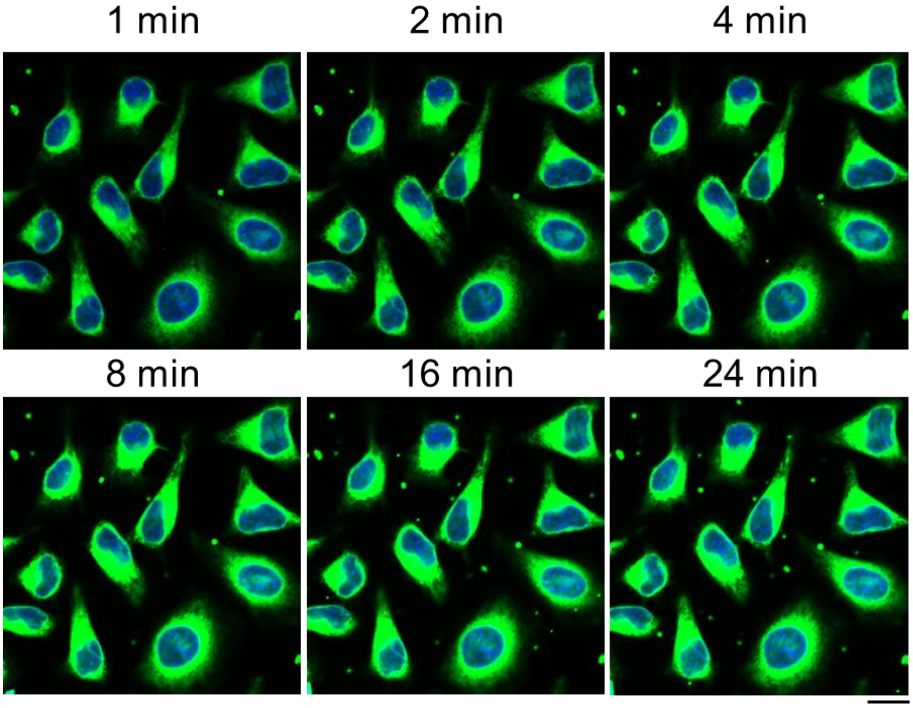

Figure S16. CLSM images of HeLa cells treated with **1p** (10 μM) over 24 minutes. Scale bar = 20 μm.

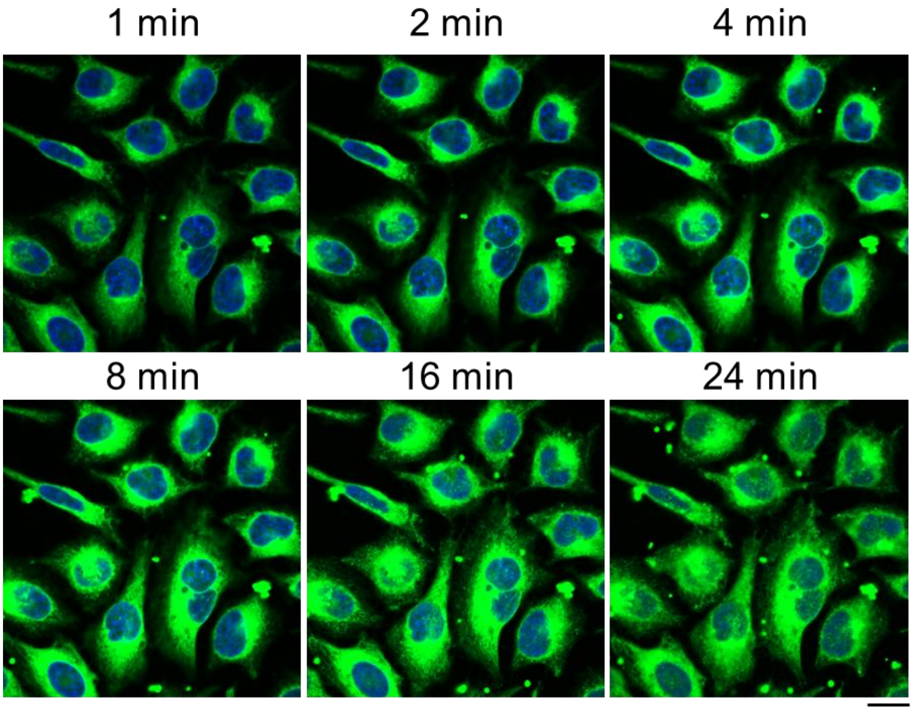

Figure S17. CLSM images of HeLa cells treated with **1q** (10 μM) over 24 minutes. Scale bar = 20 μm.

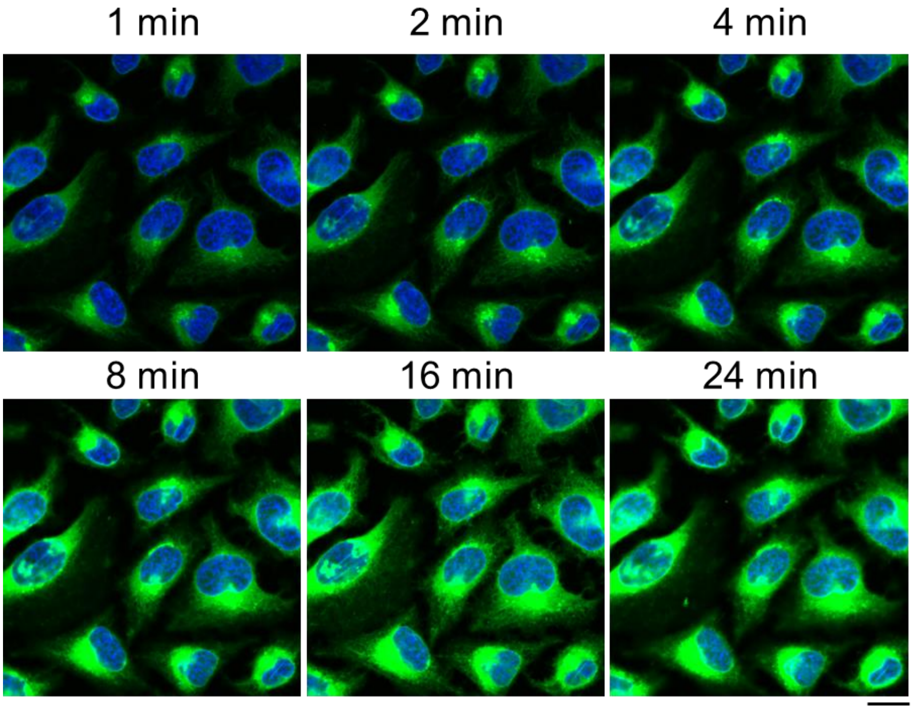

Figure S18. CLSM images of HeLa cells treated with **1v** (10 μM) over 24 minutes. Scale bar = 20 μm.

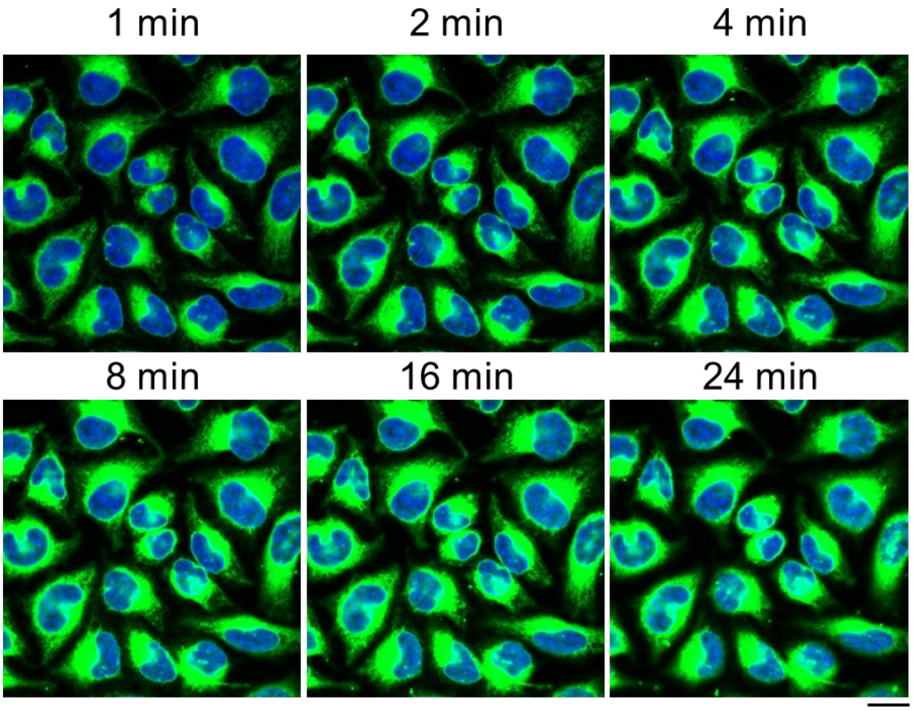

Figure S19. CLSM images of HeLa cells treated with **1w** (10 μM) over 24 minutes. Scale bar = 20 μm.

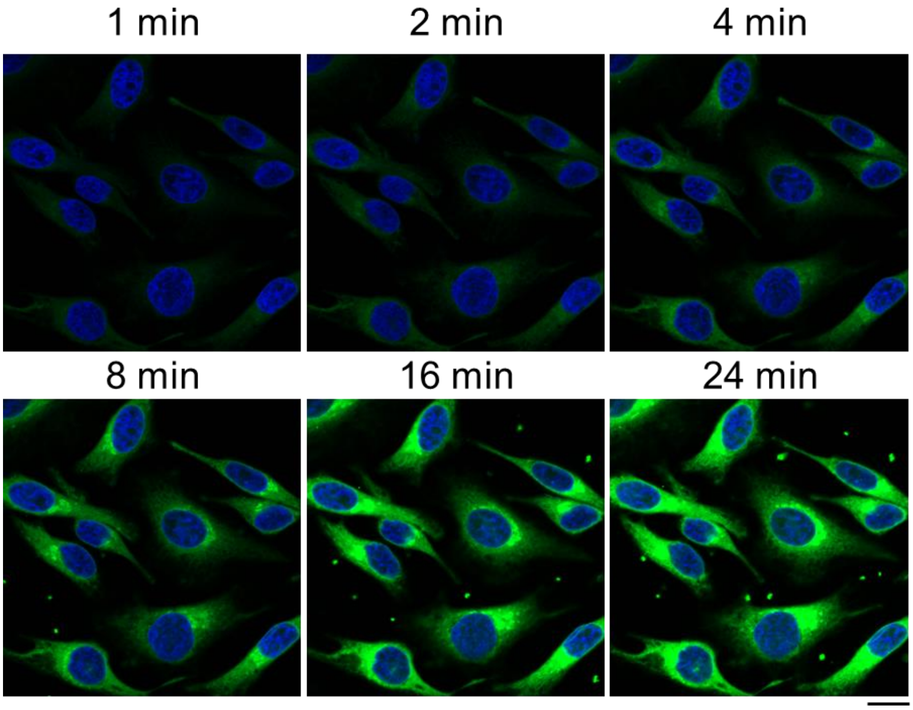

Figure S20. CLSM images of HeLa cells treated with **1u** (10 μM) over 24 minutes. Scale bar = 20 μm.

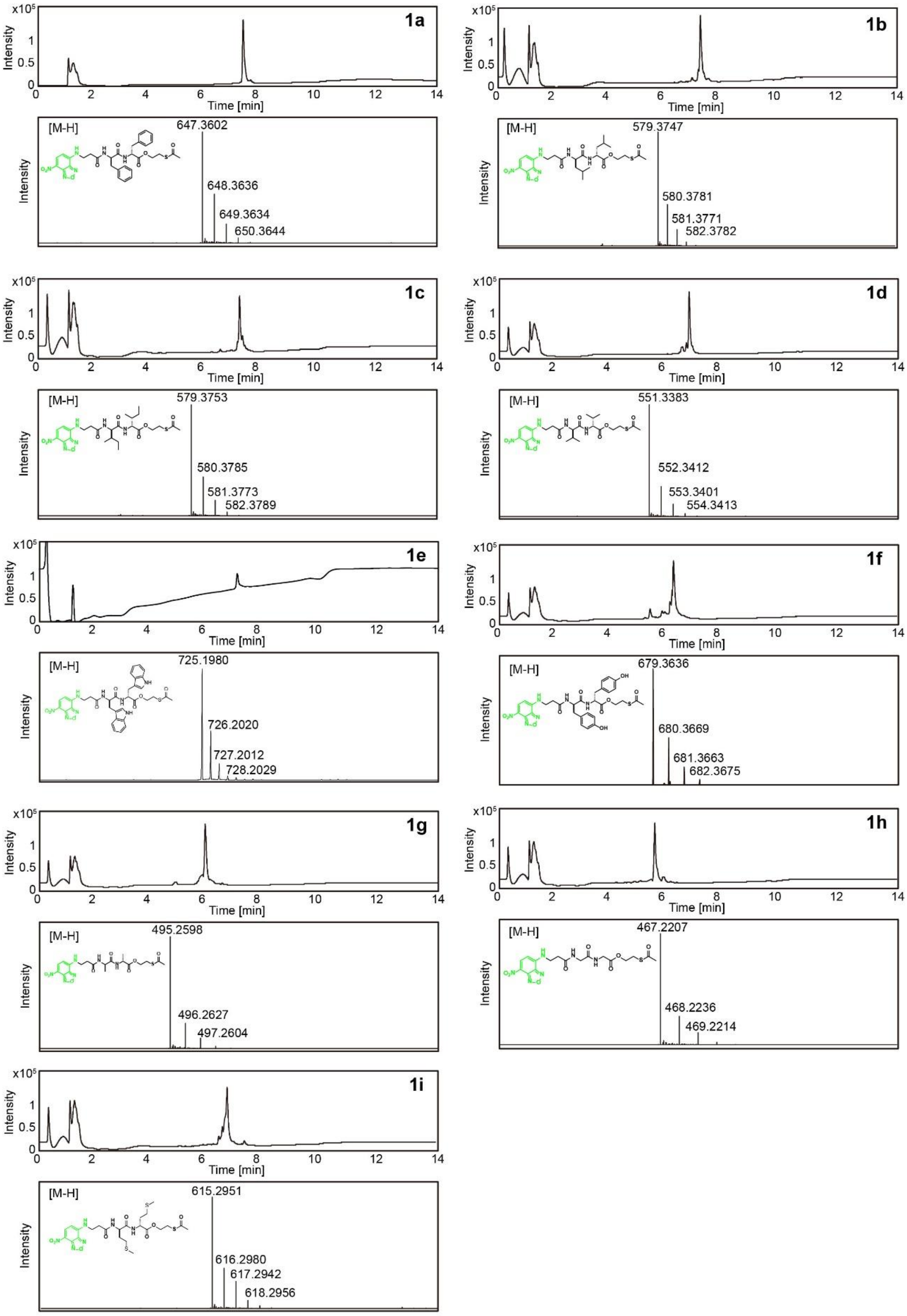

Figure S21. LC-HRMS of the synthesized **1a-1i**.

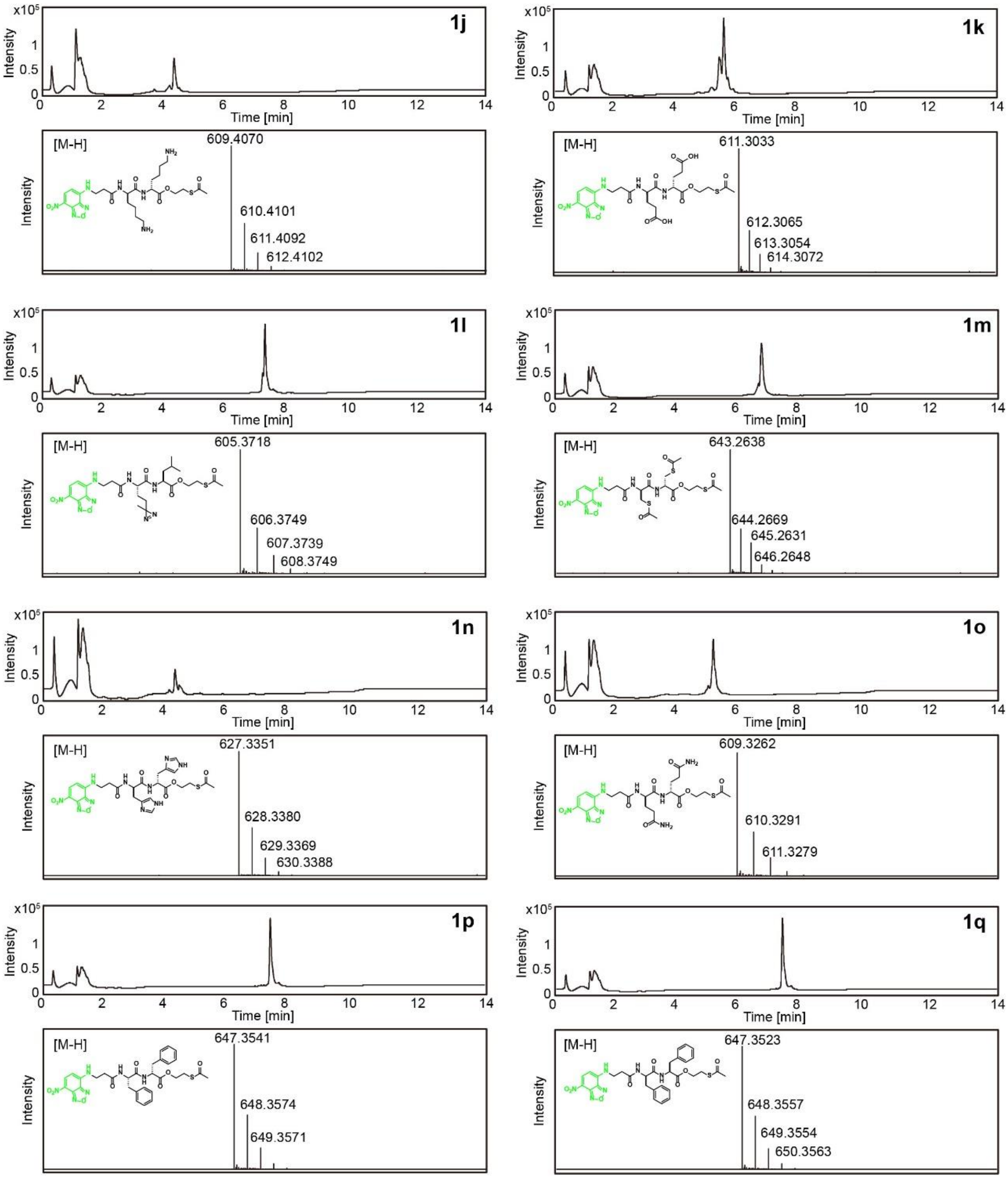

Figure S22. LC-HRMS of the synthesized **1j**-**1q**.

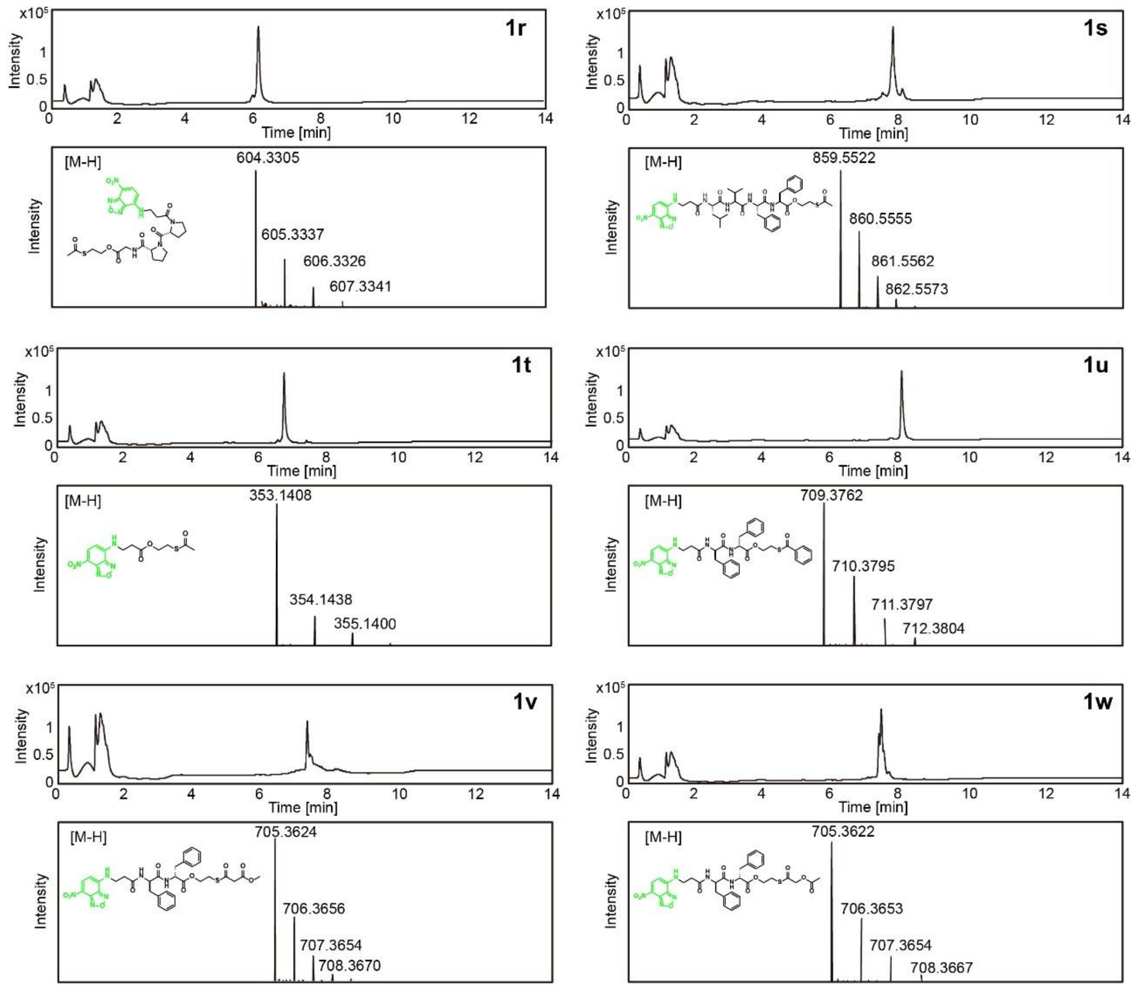

Figure S23. LC-HRMS of the synthesized **1r-1w**.

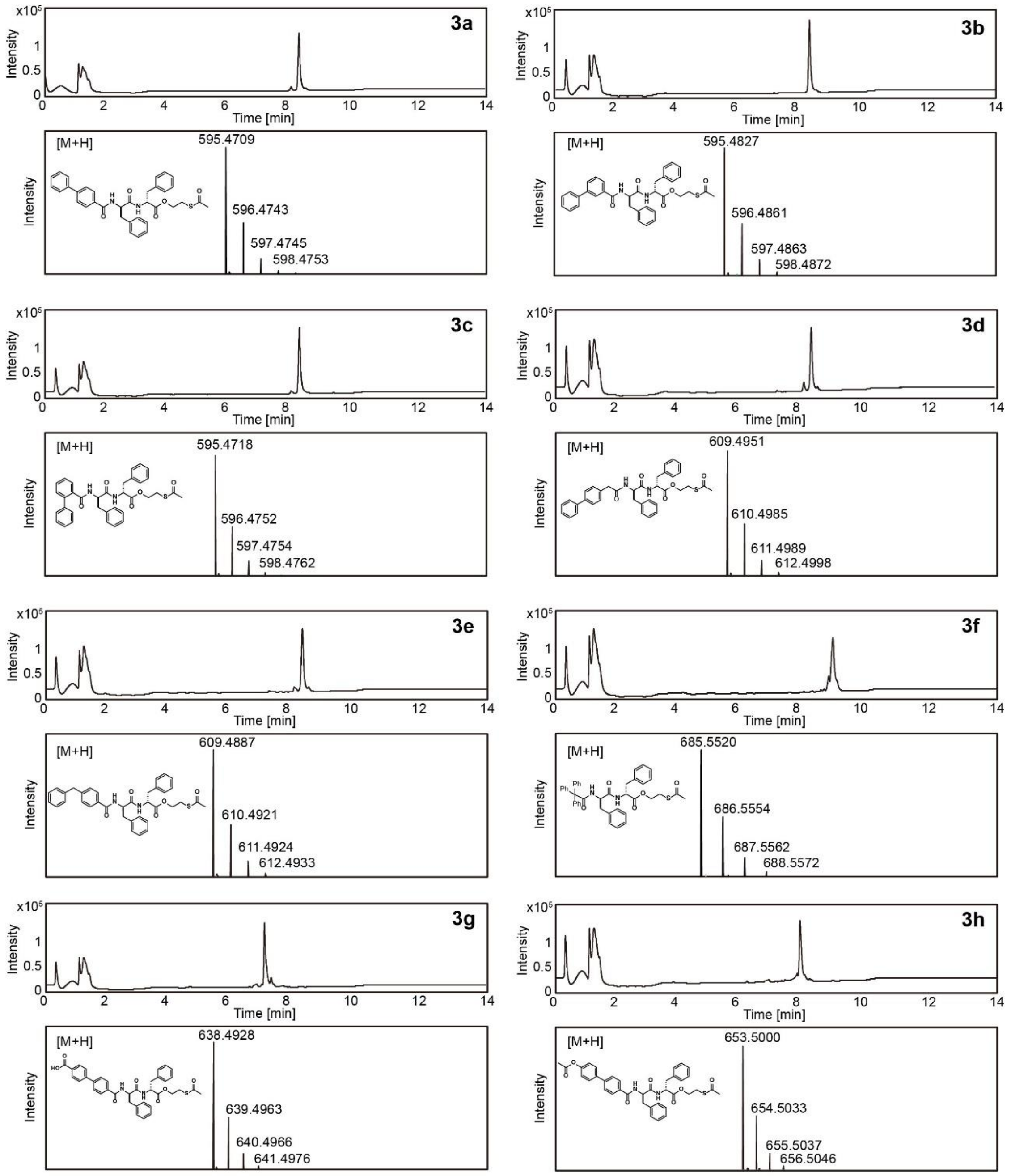

Figure S24. LC-HRMS of the synthesized **3a**-**3h**.

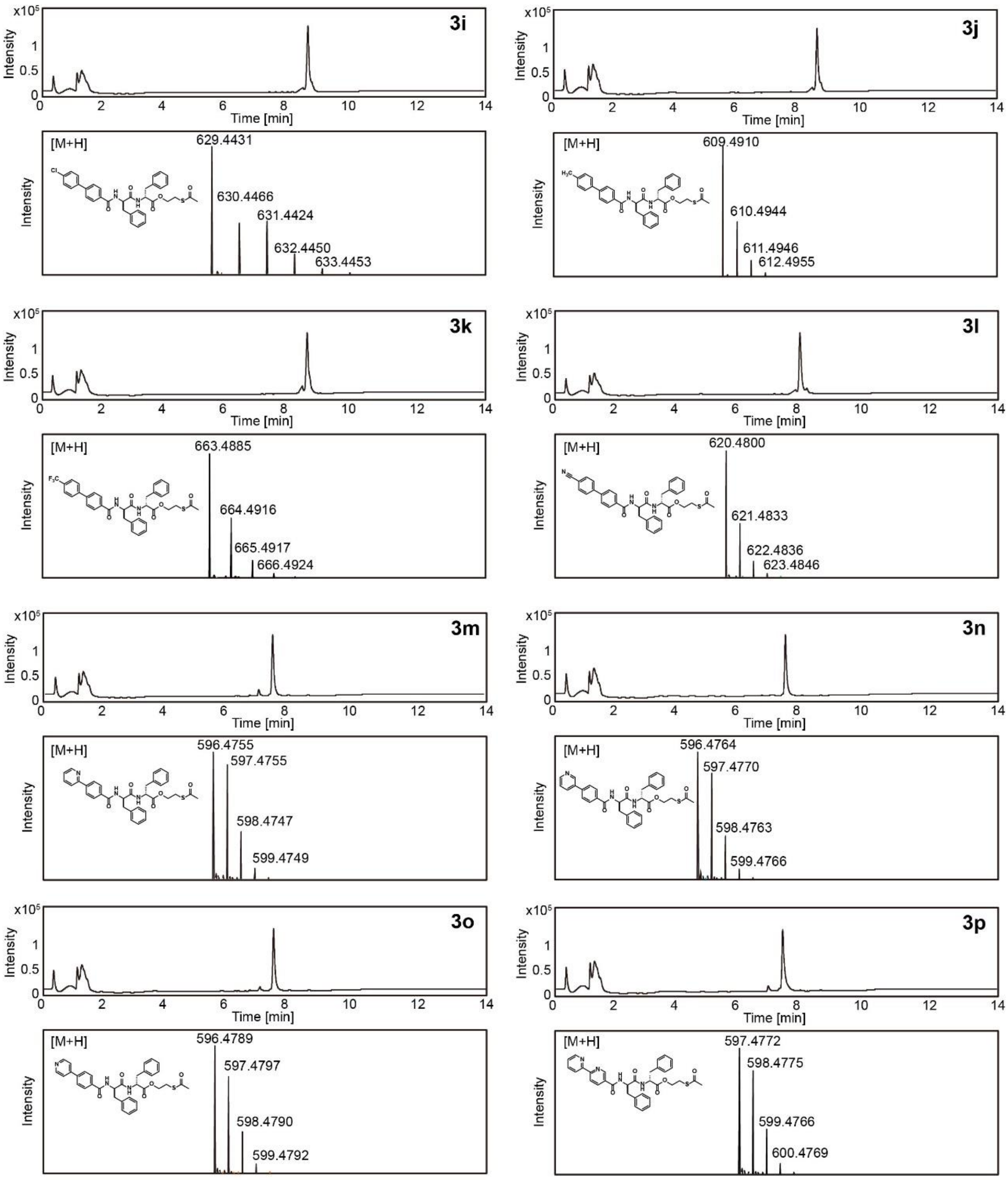

Figure S25. LC-HRMS of the synthesized **3i**-**3p**.

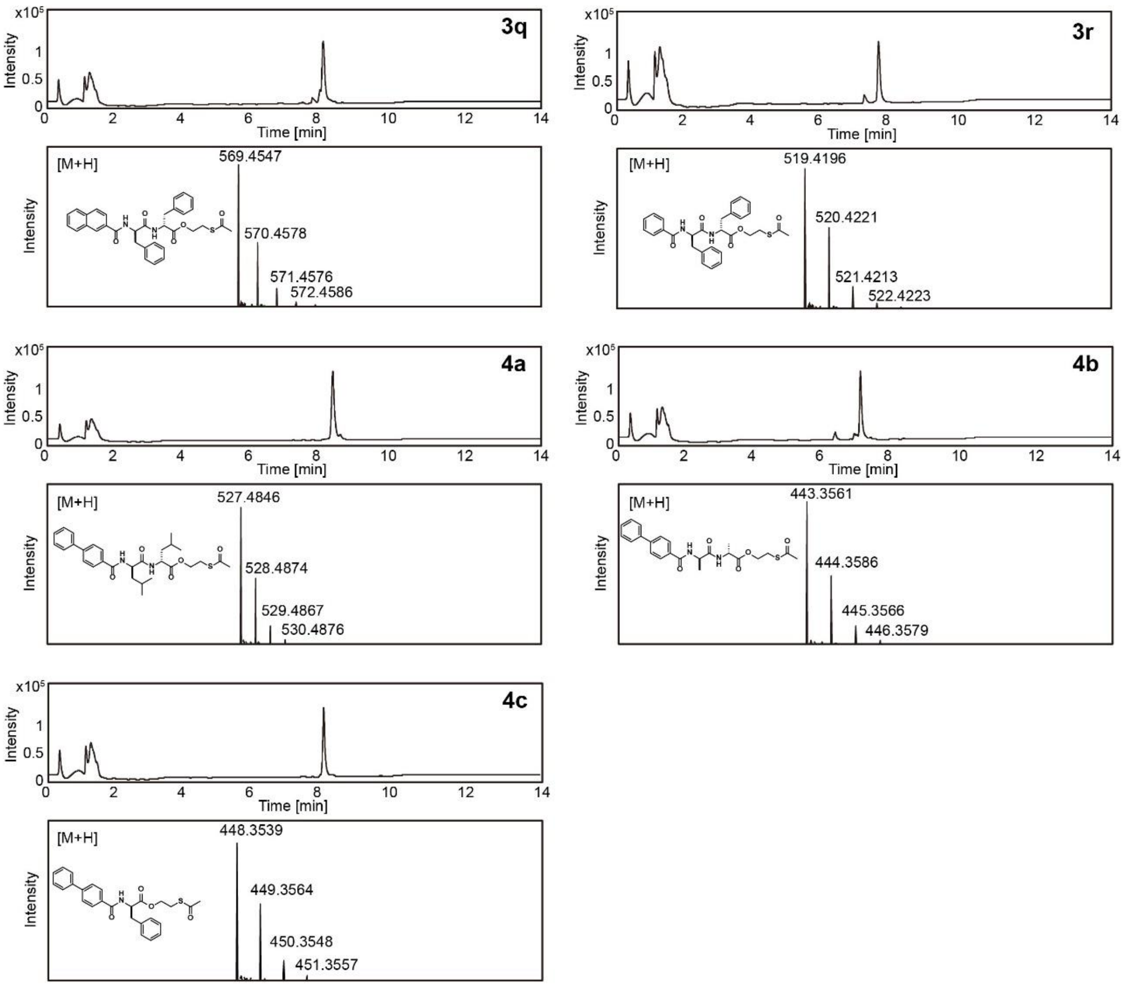

Figure S26. LC-HRMS of the synthesized **3q-3r**, **4a-4c**.

Reference

1. Tan, W.; Zhang, Q.; Quiñones-Frías, M. C.; Hsu, A. Y.; Zhang, Y.; Rodal, A.; Hong, P.; Luo, H. R.; Xu, B., Enzyme-Responsive Peptide Thioesters for Targeting Golgi Apparatus. *Journal of the American Chemical Society* **2022,** *144*, 6709-6713.

2. Joshi, V. S.; Kumar, V.; Rathore, A. S., Role of Organic Modifier and Gradient Shape in RP-HPLC Separation: Analysis of GCSF Variants. *Journal of Chromatographic Science* **2015,** *53*, 417-423.

3. Kalyanasundaram, K.; Thomas, J. K., Environmental effects on vibronic band intensities in pyrene monomer fluorescence and their application in studies of micellar systems. *Journal of the American Chemical Society* **1977,** *99*, 2039-2044.

4. Otsu, N., A Threshold Selection Method from Gray-Level Histograms. *IEEE Transactions on Systems, Man, and Cybernetics* **1979,** *9*, 62-66.
